# Molecular docking, DFT analysis, pharmacokinetic profiling and MD simulation of *Ilex aquifolium* L. flavonoids as potential α-glucosidase predicted inhibitors for diabetes

**DOI:** 10.1038/s41598-026-50123-y

**Published:** 2026-05-15

**Authors:** Afnan M. Alnajeebi

**Affiliations:** Department of Biological Sciences, College of Science, University of Jeddah, Jeddah, Saudi Arabia

**Keywords:** *I. aquifolium*, α-glucosidase, Molecular Docking, DFT Analysis, ADMET Analysis, MD Simulation, Biochemistry, Chemistry, Computational biology and bioinformatics, Drug discovery

## Abstract

The growing diabetes trend worldwide demands the identification of new α-glucosidase inhibitors for delaying postprandial hyperglycemia. However, the use of existing drugs is often limited by adverse effects, which has increased interest in natural products as safer alternative sources of α-glucosidase inhibitors. *I. aquifolium* was selected based on reports of flavonoid-rich phytochemistry, leading to the hypothesis that its major flavonoids could act as potential α-glucosidase inhibitors for the management of diabetes. Herein, the virtual α-glucosidase inhibitory potential of eight flavonoid-based phytochemicals of *I. aquifolium* extracts was investigated through an integrated in silico workflow, which includes molecular docking, DFT analysis, and ADMET profiling. Docking analysis identified IA- 2 (Rutin) and IA- 5 (Kaempferol-3-O-rhamnoglucoside) as the most potent ligands with binding affinities of − 9.59 kcal/mol and − 9.18 kcal/mol, respectively, closely approaching the standard acarbose (–10.96 kcal/mol). The re-docking RMSD of 2.1375 Å fell within the acceptable validation benchmark, supporting the reliability of the docking protocol. The second and third positions were occupied by IA- 1 (Quercetin) and IA- 3 (Quercetin-3-O-hexoside) as potential inhibitors, as they formed hydrogen bonds with hydrophobic contacts. DFT study suggested that all the flavonoids exhibited moderate to high dipole moments (4.88–7.36 Debye) along with the high HOMO–LUMO gaps. Additionally, IA- 7 (Apigenin) showed the highest electrophilicity (3.313) among all the phytochemicals. The pharmacokinetic evaluation of ligands by SwissADME and PkCSM suggested IA- 1 as the most potential hit, and it occupied the main drug-likeness parameters with a bioavailability score of 0.55, whereas low bioavailability scores (0.17) for IA- 2 and IA- 5 indicated their poor permeability through the membrane. The potential toxicity of the ligands through SwissADME predicted that all the ligands have renal and respiratory issues, but IA- 1 showed the highest potential of acute dermal toxicity. MD simulations supported the docking results by showing protein backbone RMSD stabilization at 2.0–2.4 Å and revealing that IA-2 and IA-5 form the most dynamically stable complexes (lowest ligand RMSD, compact Rg, persistent H bonds), while IA-3 and IA-1 display greater ligand flexibility, thereby prioritizing these compounds for experimental validation in a hypothesis-generating context. However, these computational findings do not replace experimental validation and should be interpreted as hypothesis-generating predictions. Overall, the study provides a hypothesis-generating framework, prioritizing IA-2 and IA-5 as potential α-glucosidase inhibitory candidates, with IA-1 emerging as a top-ranked ligand through pharmacokinetic parameters, required further experimental validation.

## Introduction

Diabetes mellitus is known to be one of the metabolic diseases characterized by hyperglycemia resulting from a deficiency of insulin^1,2^. It is a multifactorial physiological disorder that causes severe macrovascular and microvascular complications such as cardiovascular diseases, neuropathy, nephropathy, blindness, kidney failure, and mortality^3^. Diabetes was projected to affect 4.4% of the global population of all age groups by 2030^4^. Hyperglycemia of extended periods of time can stimulate the body to produce reactive oxygen species (ROS), which can cause an increase in the ROS concentration in susceptible bodily tissues such as the liver, heart, and brain, resulting in diabetic complications^5,6^. As a result, a combination of several targets of diabetes mellitus (oxidative stress and other key enzymes) becomes the major strategy in combating it^7^. Alpha glucosidase is an enzyme that has been reported to be involved in the pathogenesis of diabetes mellitus and is found in abundance in the brush borders of the small intestine. Alpha-glucosidase enzyme digests carbohydrates by selectively hydrolyzing 1→4-linked α-glucose residues and the rate-limiting disaccharide linkages, releasing single glucose molecules into the blood, thus increasing blood sugar levels^8^. Alpha-glucosidase inhibitors are known to block the enzyme’s activity by competitively binding to the enzyme, thus slowing down the digestion and absorption of carbohydrates, decreasing glucose uptake, and, as a result, preventing postprandial hyperglycemia, and this mostly occurs independent of insulin^9^. Moreover, α-glucosidase inhibitors reduce postprandial glucose excursions as an adjunctive, symptomatic therapy rather than a cure for diabetes, and therefore prioritized compounds require biochemical and in-vivo validation before clinical translation^10^. Although synthetic medication is available for the treatment of type 2 diabetes mellitus, long-term use can cause acute and life-threatening side effects such as hypoglycemia, diarrhea, and liver-related problems^9,11^. This concept is what is always required and calls for the development of new inhibitors that can possess high effectiveness with minimal toxicity^9,12^. Natural compounds such as anthocyanin-rich extract from Hibiscus rosa-sinensis flowers (HRA) were found to be inhibitors of all mammalian glucosidase activities, including maltase, sucrase, isomaltase, and glucoamylase^13^.

Flavonoids, a major subclass of plant polyphenols, are widely recognized for their potent antioxidant and free‑radical scavenging activities that protect biomolecules from oxidative damage and contribute to disease prevention^14^. Experimental HPLC/UHPLC-based phytochemical studies on *I. aquifolium* leaves and fruits have reported the presence of flavonoids such as quercetin, rutin, and related glycosides, supporting their selection in the present study^15,16^. These compounds also exert anti‑inflammatory and enzyme‑modulating effects including inhibition of carbohydrate‑digesting enzymes such as α‑glucosidase and α‑amylase supporting their relevance in antidiabetic research^17^. Moreover, flavonoids influence metabolic and gut‑microbiota pathways, offering additional mechanisms (lipid‑lowering, insulin‑sensitizing, antiglycation) that underpin their therapeutic potential and justify their prioritization in natural product drug‑discovery programs^18^.

Computer-aided drug design (CADD) approaches have dramatically revolutionized and accelerated modern drug discovery with the use of bioinformatic servers and tools, such as molecular docking^19,20^. Molecular docking is a technique that has become essential in structural biology research and is among the most commonly used methodologies for drug design^21^. Molecular docking is a computer modeling method that is used to understand and predict the interactions that occur between a small molecule (ligand) and a protein (receptor) at the atomic level^20^. The technique enables the characterization of the behavior of a small molecule within the binding site of the target protein^22,23^. Molecular docking is, in principle, comprised of two basic steps. The first step is the sampling of the conformations of the ligand into the active site of the protein^24^, and the second step is the ranking of these ligand conformations by a scoring function^21,23^. Molecular docking, in its application, is mostly employed in structure-based drug designing for the identification of new active compounds against a specific target protein^19,25^. This is because molecular docking can predict the biological activity of the phytochemicals found in a plant by predicting the phytochemicals interaction with the protein^25^. The output data, which includes the binding residues, binding energy, and inhibition constant, can be used to determine the in-vitro inhibition potential of the phytochemicals against the target protein^26^. The compounds with the lowest binding energy or highest binding affinity generally have the greatest ability to inhibit the receptor protein^26,27^. For example, the molecular docking studies were conducted to identify the inhibitory properties of phytochemicals against alpha-glucosidase^28^ and to know how well the compounds, cyanidin 3-sophoroside (CS) and quercetin 3-O-sophoroside (QS), interact with different subunits of human glucosidases^29^.

Density Functional Theory (DFT) analysis is among the molecular modeling techniques^30^. DFT calculations are usually performed to optimize the structure of a compound and determine its stability, physicochemical properties, and chemical reactivity^31^. DFT calculations are also used in the investigation of the electronic properties of molecules^32^. The analysis of the DFT calculations often involve the analysis of Frontier Molecular Orbitals (FMOs) of EHOMO and ELUMO^33^. The energy values of EHOMO and ELUMO can be used to gain insights into charge transfer in the compound^30^. A higher value of EHOMO indicates the ability of a phytochemical to donate a charge to the receptor and thus its inhibitory strength^34^. On the other hand, a lower ELUMO value indicates the strength of a molecule to receive an electron from the receptor and thus the inhibitory power of a compound^35^.

Evaluation of a compound’s potential drug profile (pharmacokinetic and toxicological properties) is very important before it can be taken further for preclinical and clinical trials^36,37^. The pharmacokinetic parameters of a compound are well evaluated through ADMET (Absorption, Distribution, Metabolism, Excretion, and Toxicity) or ADME^21^. The early detection of PK properties along with drug-likeness and ADMET profiling is important because of the fact that non-optimal bioavailability and unsuitable PK/PD properties are the leading causes of the 90% failure rate of drug candidates in clinical trials^36,38^. The ADMET properties are predicted using different computational servers and tools such as SwissADME and pkCSM^39^. Some of these parameters that are commonly evaluated and investigated include gastrointestinal absorption (GIA), Caco-2 cell permeability, blood-brain barrier (BBB) permeation, plasma protein binding, metabolism by cytochrome P450 (CYP) enzymes, and some toxicity endpoints^40^. Evaluation of drug-likeness is also important and is mostly done by using Lipinski’s Rule of Five (Ro5), where properties such as molecular weight, lipophilicity (logP), and the number of hydrogen bond donors and acceptors are determined^41,42^. The overall analysis is to confirm the relevancy and safety of the potential drug candidates, thus allowing researchers to conserve time and resources that would have been used in drug discovery^21,36^. Toxicity prediction is also commonly performed with the help of servers such as ProTox-II and ProTox-3.0 by evaluating certain endpoints such as hepatotoxicity and carcinogenicity^43–45^.

Hyperglycemia management with potent but less toxic alpha-glucosidase inhibitors has created a high demand for the urgent use of plant-derived compounds, especially flavones and flavonols, in the management of diabetes^9^. Although a number of complex and advanced computational approaches such as molecular docking and ADMET profiling are widely and commonly used to quickly screen and validate the drug-likeness of natural inhibitors^12,39^, only a few of such comprehensive studies have been documented and most of them lack the employment of DFT calculations to help further characterize and determine the electronic stability and chemical reactivity^31,33^. Molecular dynamics (MD) simulation is a powerful computational approach used to investigate the dynamic behavior and stability of biomolecular systems at the atomic level. While molecular docking predicts the preferred binding orientation of ligands within a protein’s active site, MD simulations provide deeper insight into the conformational flexibility and time-dependent stability of protein–ligand complexes under physiological conditions^46^. By applying Newton’s equations of motion, MD simulations enable the exploration of structural fluctuations, interaction patterns, and binding stability over time, making them an essential tool in modern structure-based drug discovery^47^. Therefore, because no prior study has combined molecular docking, DFT, ADMET and MD simulation for *I. aquifolium* flavonoids, we applied an integrated in‑silico workflow to generate electronic and dynamic profiles that prioritize IA‑2 and IA‑5 for targeted in‑vitro validation.

## Methodology

The computational workflow, including ligand and protein preparation, molecular docking, DFT analysis and ADMET profiling, was used to predict the activity of *I. aquifolium* flavonols and flavones as alpha-glucosidase inhibitors.

### Ligand and target preparation

The ligand structures were retrieved from chemical databases such as the Indian Medicinal Plants Phytochemistry and Therapeutics (IMPPAT) (https://cb.imsc.res.in/imppat/home) database; however, these resources were used solely for compound retrieval, while flavonoid selection was guided by literature reports of *Ilex aquifolium* (e.g., quercetin and rutin derivatives) and by chemotaxonomic relevance within the genus. The crystal structure of the target protein, alpha-glucosidase (PDB ID: 5NN8), was obtained from the PDB in PDB format with resolution of 2.45 Å^48^. The 5NN8 was selected because of in-bound inhibitor (acarbose) in active site, enabling us to dock our ligand in the same pocket and compare the results with the known inhibitor for a more accurate evaluation of potential α-glucosidase inhibition. The standard reference inhibitor, acarbose, was also procured^29^. Molecular interactions of the standard inhibitor, acarbose and the four top-scoring docked molecules were visualized and analyzed using BIOVIA Discovery Studio Visualizer in order to observe and characterize key intermolecular interactions, including conventional hydrogen bonding, hydrophobic interactions, sulfur and electrostatic interactions between the ligands and the catalytic residues within the binding pocket^49^.

### Molecular docking

#### Ligand preparation (LegPrep)

The ligands were prepared using the Ligprep module (Maestro, Schrödinger) using coordinates for the molecules and generates stereoisomers for the prepared molecules and it also corrects tautomeric states and energy minimized using the Optimized Potentials for Liquid Simulations (OPLS) force field^29^.

#### Protein preparation

The protein was prepared using Protein Preparation Wizard in Schrödinger 2020-3 (Maestro 12.5). The preparation involves deleting non-amino acid residues such as waters and heteroatoms and addition of hydrogens atoms to the protein, which is then minimized to improve the protein structure and geometry^42^.

#### Ligand docking

The molecular docking was performed with the prepared ligands and the prepared -glucosidase receptor (5NN8) using Schrödinger 2020-3 (Maestro 12.5). Ligands were prepared using LigPrep with the OPLS3e force field and protonation states generated at pH 7.0 ± 2.0 using Epik. Tautomers were generated, and only 1 isomer per ligand was retained. Ligands with more than 500 atoms or 100 rotatable bonds were excluded, and their Van der Waals radii were scaled by a factor of 0.8 with a 0.15 partial charge cutoff. The protein structure was preprocessed with Protein Preparation Wizard, adding hydrogens, assigning bond orders, and filling missing side chains and loops using Prime. Heteroatom states were generated with Epik at pH 7.0 ± 2.0. The molecular docking was performed using the Ligand Docking module with extra precision (XP) mode to compute the optimal binding poses of the ligands. For docking, the receptor grid was set up in Glide XP (Extra Precision) with a grid size of 80 × 80 × 80 and the center defined by the centroid of selected residues. The Glide receptor grid was centered at coordinates X = − 14.0336, Y = − 38.2028, Z = 95.1932 (centroid of the co-crystallized ligand site). The grid geometry used an INNERBOX = 10 Å, OUTERBOX = 28.6014 Å (equivalent to a grid of 80 × 80 × 80 points); receptor VdW scaling = 1.00, ligand VdW scaling = 0.80 with a 0.15 partial-charge cutoff. Only ligands smaller than 20 Å were included, and the OPLS3e force field was used. 10 poses per ligand were retained, post-minimized with a 0.50 kcal/mol rejection threshold. Epik state penalties were applied to account for protonation states, and docking was performed with the GlideScore XP5.0 scoring function. The binding site grid box was generated around the active cavity of the enzyme based on the presence of the co-crystallized ligand or the catalytic residues. The main output is the docking score that is expressed in terms of binding affinity, and the molecules are ranked according to their binding energy; compounds with the lowest binding energy are selected^29^.

### DFT analysis

Density functional theory (DFT) calculations were performed to study the electronic and quantum chemical properties of the best-docked ligands^32^. DFT calculations were used to optimize the molecular structure and to compute the energies of the Highest Occupied Molecular Orbital (HOMO) and the Lowest Unoccupied Molecular Orbital (LUMO), Molecular Electrostatic Potential (MESP). The DFT parameters such as the energy values (e.g. in eV), were calculated in a solvent environment using a solvation model^30^. The and values yield important insights into the chemical reactivity and charge transfer ability of the molecules and to support the molecular docking results^34^. The DFT calculations were carried out using the B3LYP/3-21G level of theory with the C-PCM solvation model, where water was used as the solvent. The calculations ensured convergence of the density matrix (RMSD threshold of 1.00D-08), and frequency calculations confirmed the true minima of all optimized structures with no imaginary frequencies.

### ADMET and toxicity prediction

Computational pharmacokinetic and drug-likeness profiling was performed, as the early prediction of these parameters is important to validate drug candidates^38^. The ADMET properties (gastrointestinal absorption, Lipinski’s Rule of Five, bioavailability) were predicted with the web server SwissADME (https://swissadme.ch/)^39^. In addition to this, a series of ADMET parameters (Caco-2 cell permeability and inhibition of Cytochrome P450 (CYPs) enzymes) were predicted with the web server PkCSM (https://biosig.lab.uq.edu.au/pkcsm/)^49^. Toxicity profiling was performed using specialized online tools, including ProTox 3.0 (https://tox.charite.de/protox3/index.php?site=compound_input) and STopTOX (https://stoptox.mml.unc.edu/)^45^. These tools predict various toxicity endpoints, such as hepatotoxicity, carcinogenicity, mutagenicity, and acute oral toxicity, using the canonical SMILES string of the compounds as input^42^.

### Molecular dynamic simulation (MD simulation)

Molecular dynamics (MD) simulations were performed for 100 ns using the Desmond module of Schrödinger, LLC^50^. Prior to MD simulation, molecular docking was carried out as an essential preliminary step to predict the most favorable binding orientation of each ligand within the active site of the target protein, thereby providing a static view of the protein–ligand interaction^51^. Based on the docking results, the top-scoring docked complexes of IA-2, IA-5, IA-3, and IA-1 were selected for further MD analysis. The selected protein–ligand complexes were prepared using Maestro’s Protein Preparation Wizard, which involved structural optimization, energy minimization, and the addition of missing residues where necessary. System setup was subsequently performed using the System Builder tool. An orthorhombic simulation box was generated, and the system was solvated using the TIP3P (Transferable Intermolecular Potential with 3 Points) water model. The simulations were conducted under NPT conditions at 300 K and 1 atm using the OPLS_2005 force field^51^. OPLS_2005 was selected for MD simulations due to its established reliability in long-timescale protein–ligand stability studies, while OPLS3e was used during docking as part of the Glide protocol. To maintain electrostatic neutrality, appropriate counterions were added, and 0.15 M sodium chloride was included to mimic physiological ionic strength. Before the production run, each system was relaxed using the default Desmond relaxation protocol. During the simulation, trajectories were recorded at intervals of 100 ps for subsequent analysis. By applying Newton’s classical equations of motion, MD simulations enable the assessment of atomic movements over time and provide valuable insight into the stability and binding behavior of ligand–receptor complexes under physiological conditions^52^.

All results are computational predictions subject to force-field and scoring-function limitations inherent to the employed software and should be validated experimentally.

## Results and discussion

### Identification and classification of phytoligands

The identified phytochemicals (IA- 1 to IA- 8) from the IMPPAT database, with a focus on antioxidant activities, were retrieved as the lead for further studies due to their significant importance in countering the oxidative stress that is associated with diabetes^53^. We have selected the eight molecules for this study, and they all belong to the flavonoid class of phytochemicals (Table 1). This includes six flavonols (IA- 1: Quercetin; IA- 2: Rutin; IA- 3: Quercetin-3-O-hexoside; IA- 4: Kaempferol; IA- 5: Kaempferol-3-O-rhamnoglucoside; IA- 6: Isorhamnetin) and two flavones (IA- 7: Apigenin; IA- 8: Luteolin). The structures, both glycosylated as well as aglycone forms, of these *I. aquifolium* flavonoids were further subjected to the integrated in silico approach molecular docking, DFT analysis, and ADMET profiling for a detailed study of their antidiabetic efficacy against α-glucosidase (5NN8).

The selection of these flavonoids is rationalized on the basis of the dual therapeutic approach necessary to address the diabetic condition by α-glucosidase inhibition and oxidative stress reduction^29^. In particular, the aglycone compounds, the flavonols Quercetin (IA- 1) and Kaempferol (IA- 4), have a known high capability of radical scavenging as evidenced by their activities against DPPH, ABTS, and superoxide assays, in addition to having high ferric reducing antioxidant power (FRAP)^54^. The flavonoids’ antioxidant activity is found to be influenced by the chemical structure of the flavonoids, and is linked to a distinct structural feature, such as the o-dihydroxyl group of the B-ring, which contributes to the free radical scavenging ability of the flavonoids^42,55^. In a similar pharmacological study, phytochemicals, specifically flavonoids and phenolics, were shown to have strong antioxidant potential and target proteins involved in key chronic diseases^56^. Anthocyanin-rich extracts containing structurally similar flavonoids to IA- 1 and IA- 4, such as cyanidin 3-sophoroside and quercetin 3-O-sophoroside, for example, have shown considerable in vitro antioxidant activity and mammalian glucosidase inhibitory activity^29^. Additionally, other research studies investigating the active ingredients of different plants, such as *Feijoa sellowiana*, have also employed several in vitro antioxidant assays (DPPH, ABTS, FRAP, CUPRac) and molecular docking to validate a combined inhibitory and antioxidant activity of identified compounds such as Quercetin^28^. Here, with the help of an integrated in silico approach, a prediction can be made in order to bridge the flavonoids of *I. aquifolium* known antioxidant potential and their mechanistic mode as α-glucosidase inhibitors.

**Table 1:** Shows a comprehensive list of *I. aquifolium* flavonoids with 2D and 3D structures as well as SMILES notation.

Code	Comp Name	2D Structure	3 D Structure	SMILES
IA- 1	Quercetin			OC1 = C(C2 = CC = C(O)C(O)=C2)OC3 = C(C(O) = CC(O)=C3)C1 = O
IA- 2	Rutin (Quercetin-3-O-rutinoside)			OC1 = CC(O)=CC2 = C1C(C(O[C@@H]3[C@@H](O)[C@H](O)[C@@H](O)[C@H](CO[C@@H]4[C@@H](O)[C@@H](O)[C@H](O)[C@@H](C)O4)O3) = C(C5 = CC(O) = C(O)C = C5)O2) = O
IA- 3	Quercetin-3-O-hexoside			O=C1C(OC2C(C(C(C(CO)O2)O)O)O) = C(C3 = CC = C(O)C(O)=C3)OC4 = C1C(O) = CC(O)=C4
IA- 4	Kaempferol			OC1 = C(C2 = CC = C(O)C = C2)OC3 = C(C(O) = CC(O)=C3)C1 = O
IA- 5	Kaempferol-3-O-rhamnoglucoside			CC1C(C(C(C(O1)OC2 = CC(= C3C(= C2)OC(= C(C3 = O)OC4C(C(C(C(O4)CO)O)O)O)C5 = CC = C(C = C5)O)O)O)O)O
IA- 6	Isorhamnetin			COC1 = C(C = CC(= C1)C2 = C(C(= O)C3 = C(C = C(C=C3O2)O)O)O)O
IA- 7	Apigenin			C1 = CC(= CC=C1C2 = CC(= O)C3 = C(C = C(C=C3O2)O)O)O
IA- 8	Luteolin			C1 = CC(= C(C=C1C2 = CC(= O)C3 = C(C = C(C=C3O2)O)O)O)O

### Protein retrieval and pocket analysis

**Fig. 1:**
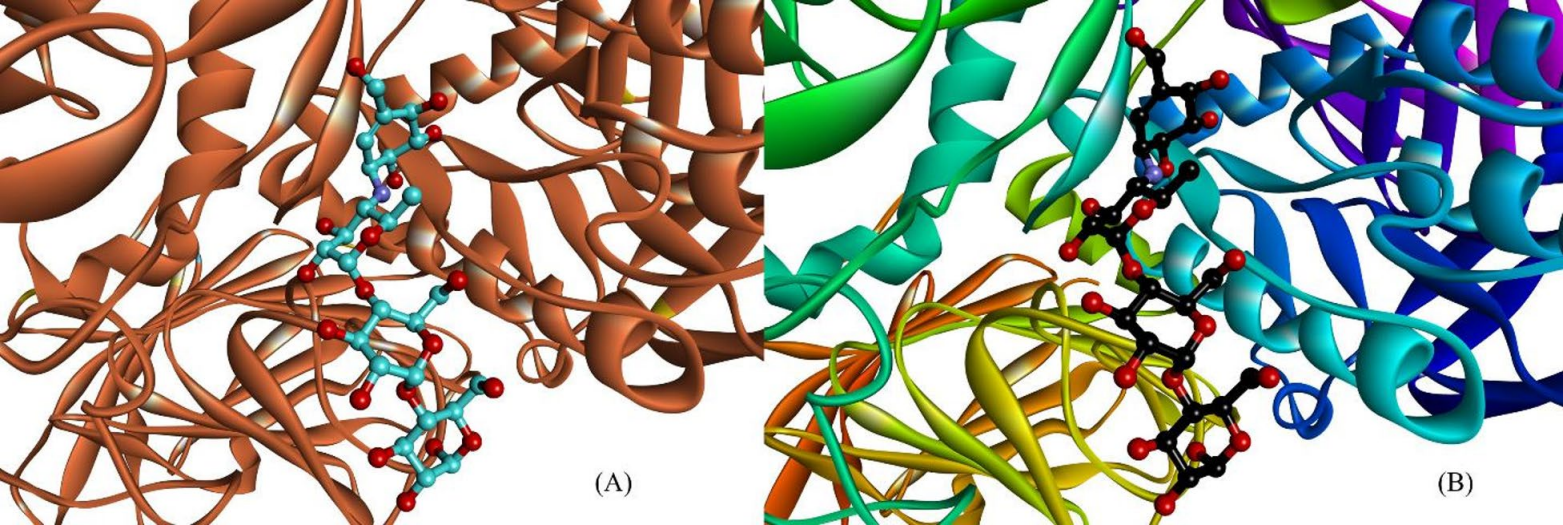
**(a**,** b)** Depicts the two orientations of α-glucosidase (PDB ID: 5NN8) that accommodate the standard inhibitor acarbose on the protein surface.

**Fig. 2:**
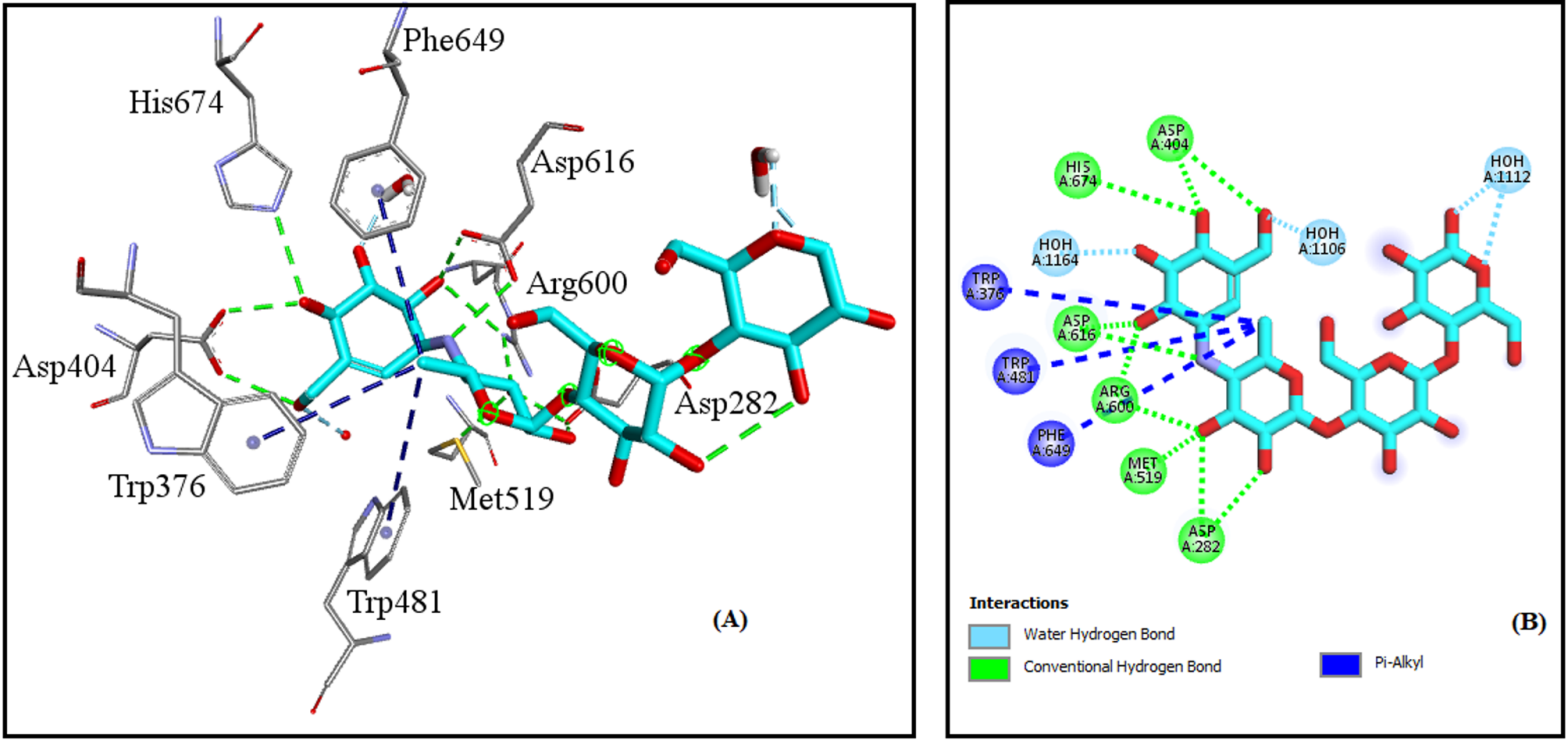
Show the two- and three-dimensional visualization of α-glucosidase (PDB ID: 5NN8) in complex with acarbose, revealing the varied interaction profiles, spatial orientation, and bonding patterns that stabilize the enzyme–inhibitor interaction.

**Fig. 3:**
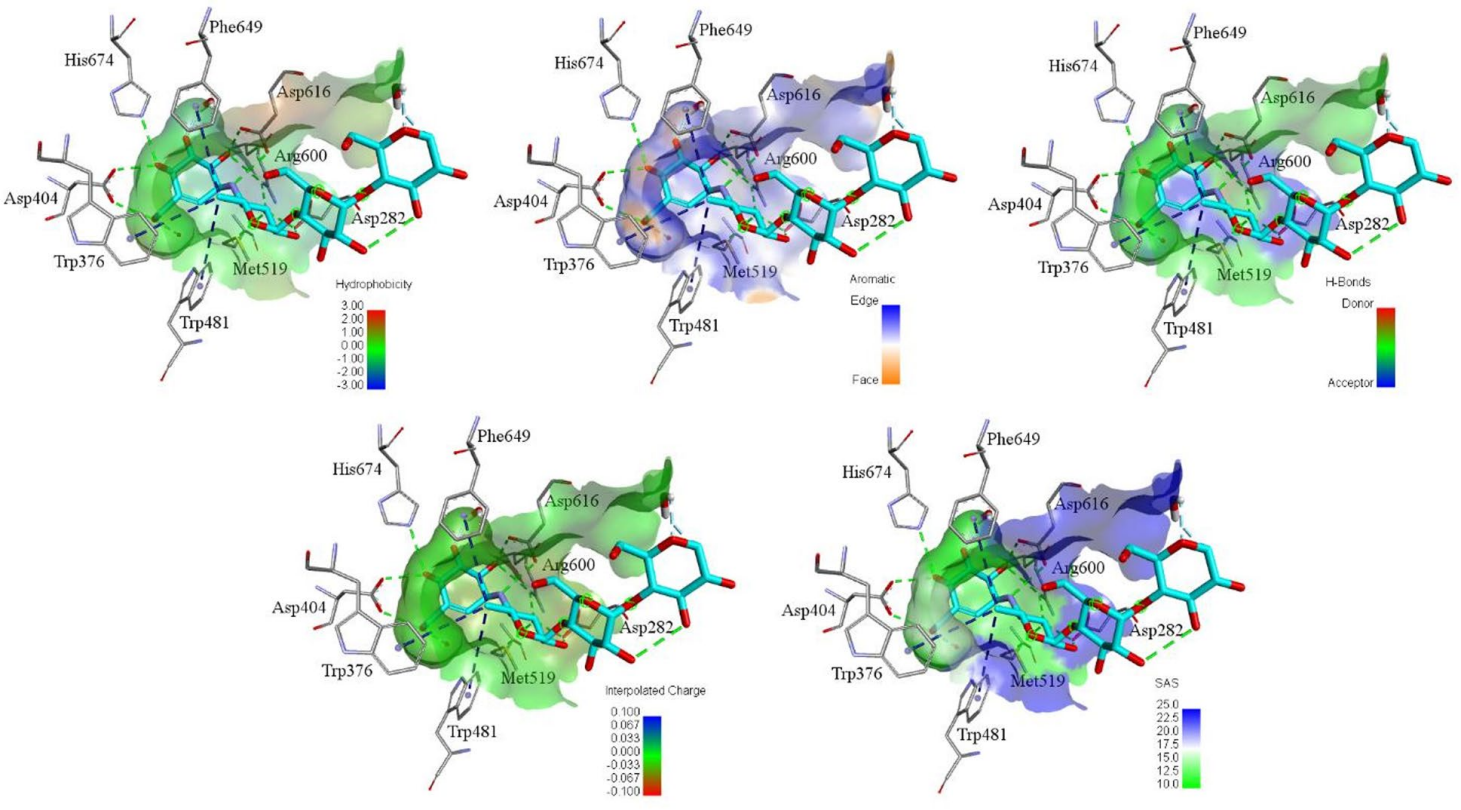
Analysis of the G-pocket region of α-glucosidase (PDB ID: 5NN8) showing hydrophobicity, aromaticity, H-bonds, charge, solvent-accessible surface (SAS), and molecular interaction distribution within the binding region.

**Fig. 4:**
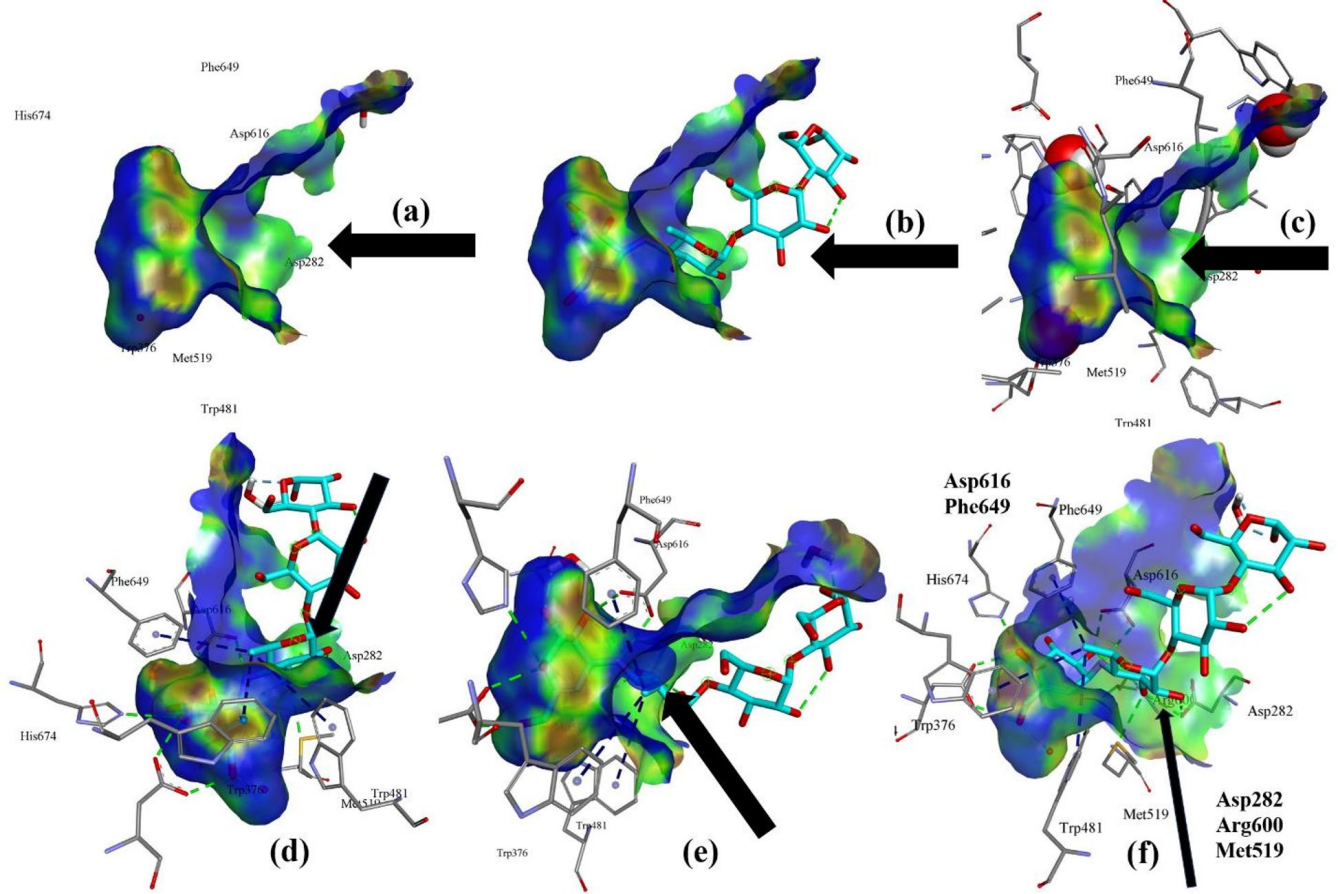
Structural visualization of the entrance channel of α-glucosidase (PDB ID: 5NN8) complexed with the standard inhibitor acarbose, illustrating multiple perspectives of the binding pocket and key amino-acid residues governing substrate entry and recognition.

The first computational step of visualizing the complex structure of the standard inhibitor of α-glucosidase enzyme, acarbose, which was retrieved from the PDB database using PDB ID: 5NN8, has been shown in Fig. 1. The standard inhibitor is deeply buried within the active site to support its well-established role as a competitive inhibitor of the carbohydrate hydrolysis by the enzyme. The complex interaction 3D profile (Fig. 2a) shows that acarbose stability depends on a large number of non-covalent interactions, including a good number of Conventional Hydrogen Bonds, Water Hydrogen Bonds, and Pi-Alkyl contacts. A few of the residues of the active site that the ligand is hydrogen bonded to directly are Asp404, Asp282, and also a water (HOH A:1164) and another water (HOH A:1106) molecule. Pi-Alkyl contacts with hydrophobic aromatic residues such as Trp376, Trp481, and Phe649 are shown in Fig. 2b. Surface Analysis of the binding cavity from the various views reveals the different physicochemical environments of the α-glucosidase binding pocket. A Hydrophobicity map has been shown in Fig. 3a, where the range is from − 3.00 (hydrophilic and shown as blue) to 3.00 (hydrophobic and shown as red). The pocket has a mixed polar/non-polar environment that is favorable for substrate recognition. Interpolated Charge map in the range of approximately − 0.100 (red color, negative charge) to 0.100 (blue color, positive charge) has been shown in Fig. 3b. The negative charged pocket and positive charged residues are found to be in close vicinity with each other along with catalytic amino acids, such as Aspartate residues, may also be included^57^. In addition, spatial visualization from different views is also observed, as shown in Fig. 4a to f, and can be used to conclude that acarbose is in a specific position in the binding channel, as the critical stabilizing interactions found between the ligand and residues such as Asp616, Arg600, and Met519 which also lines the pocket.

The presence of a stable complex of acarbose with the α-glucosidase active site is consistent with the fact that acarbose is a known competitive inhibitor of α-glucosidase, since it directly targets key catalytic and substrate-binding residues^58^. The presence of a significant number of hydrogen bonds, in particular, involving key catalytic Aspartic acid residues involved in the enzyme mechanism is also in line with the highly conserved nature of α-glucosidase active site across various mammalian enzymes^29^. It is also well-known that a higher number of conventional hydrogen bonds, as well as water-mediated hydrogen bonds, is a signature of strong binding of the ligand to the macromolecule and therefore the stable complex structure^49^. Moreover, the involvement of aromatic residues such as Trp and Phe to the hydrophobic and π-alkyl interactions between the enzyme and acarbose is also known to greatly contribute to the increased binding affinity of the ligand and to geometric stabilization of the docked complex^27^. These stabilizing interactions, in particular, the Van der Waals and electrostatic energies between acarbose and the enzyme active site, can be major contributors to the overall negative binding free energy that can be calculated with methods such as MM/GBSA^30^. Therefore, use of this well-defined interaction profile of acarbose can help in benchmarking the ability of the novel phytochemical inhibitors to engage comparably well with the enzyme’s catalytic machinery^59^.

### Molecular docking

**Fig. 5:**
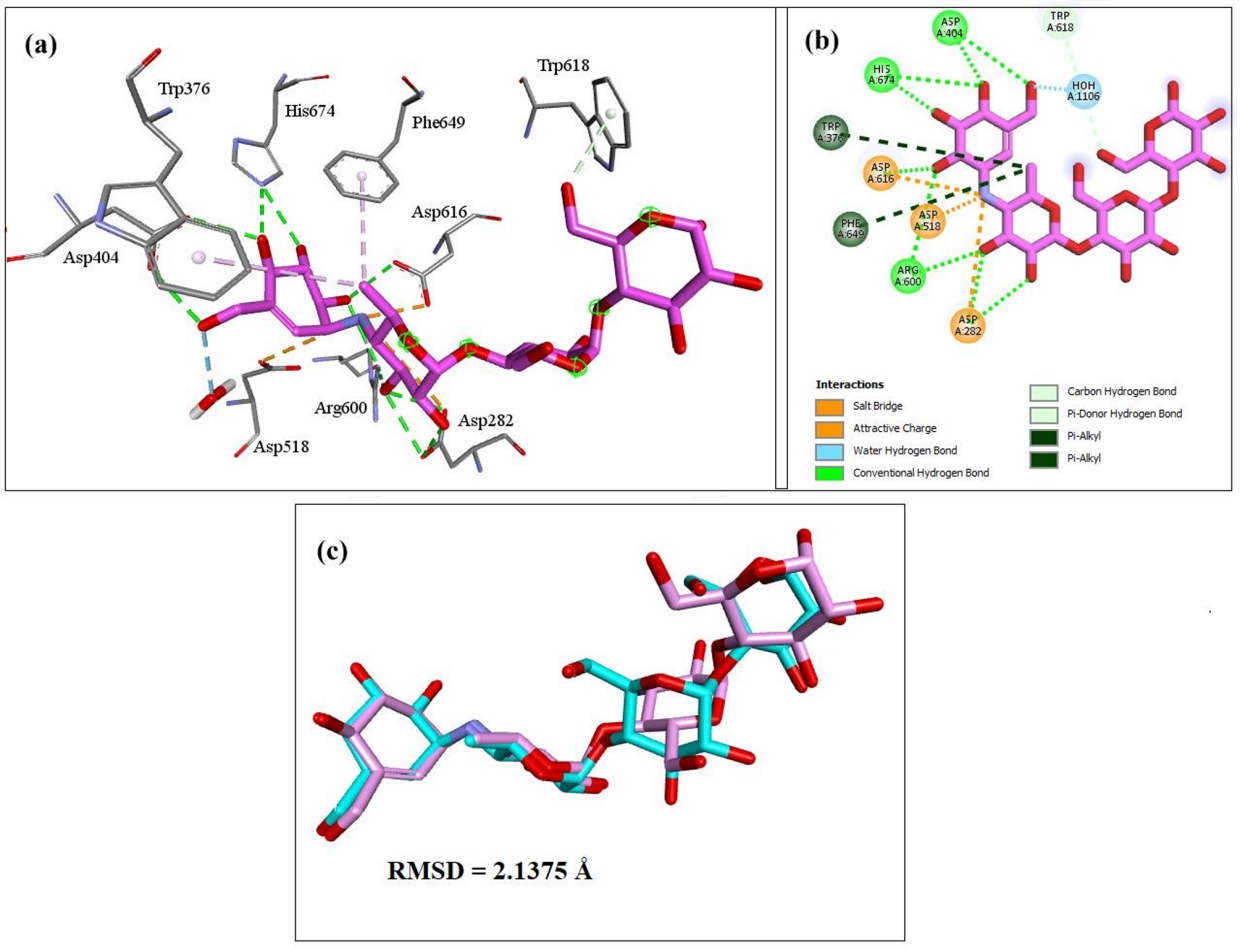
2D (**a**) and 3D (**b**) views of the docking interaction between acarbose and α-glucosidase (ID 5NN8), together with structural superposition (**c**) showing conformations before (cyan) and after (purple) docking.

**Fig. 6:**
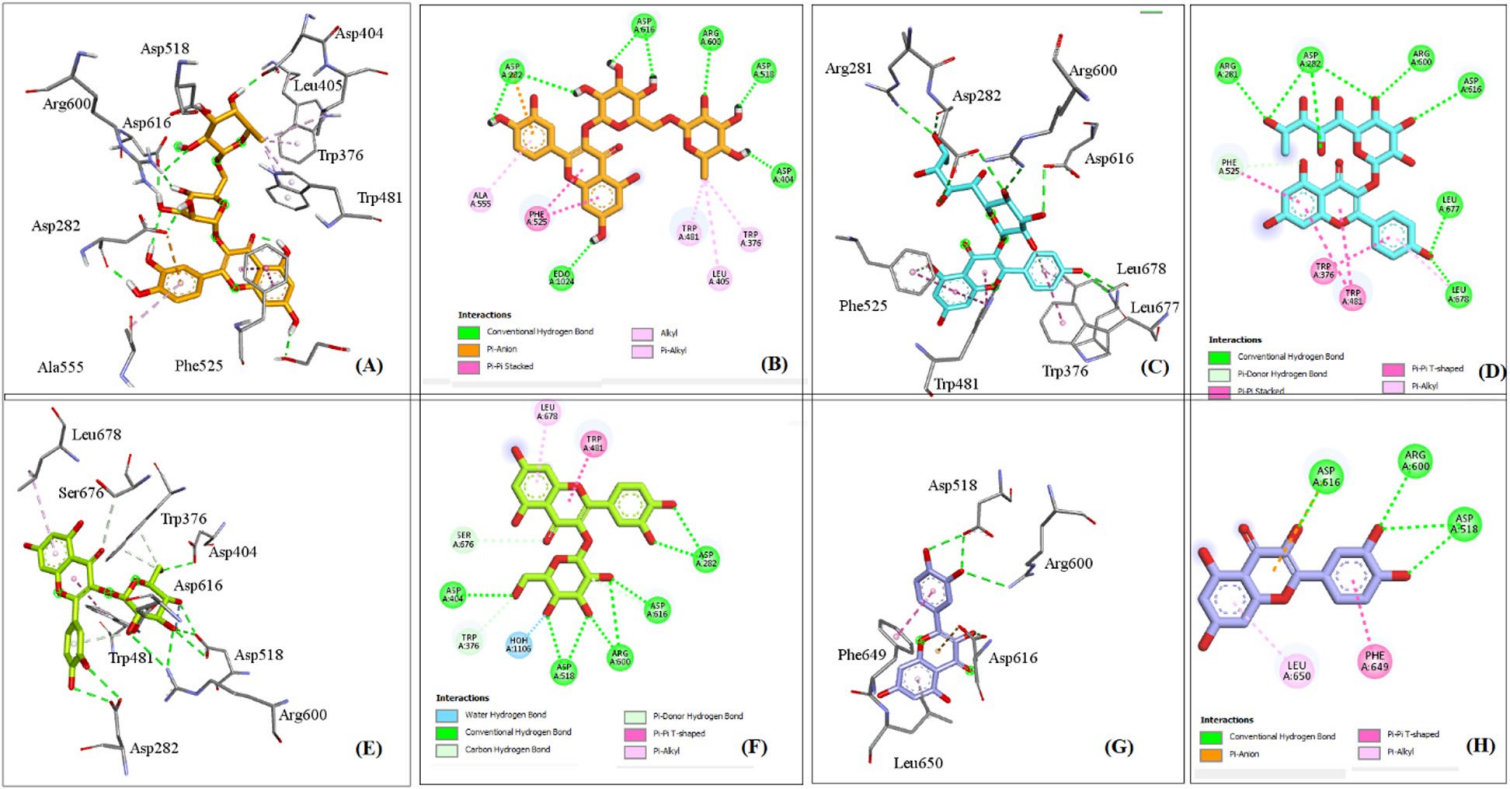
3D and 2D visualization of ligand–enzyme interactions of IA- 2, IA- 5, IA- 3, and IA- 1 with α-glucosidase (PDB ID: 5NN8) after molecular docking, highlighting key hydrogen bonds, hydrophobic contacts, and spatial conformations that define binding affinity and pocket complementarity.

Docking of *I. aquifolium* phytoligands with α-glucosidase (PDB ID: 5NN8) have been carried out, and ligands showed promising binding affinity (Table 2). The top two best scoring docked poses were of IA- 2 (–9.59 kcal/mol) and IA- 5 (–9.18 kcal/mol) closely followed by the third (IA- 3: − 6.81 kcal/mol) and fourth best scoring IA- 1 (–6.45 kcal/mol) as against the standard inhibitor of α-glucosidase, acarbose (–10.96 kcal/mol). The superposition of acarbose pose pre- and post-docking was performed and observed an RMSD value of 2.1375 Å, and is a good indication of stability for the generated docking poses (Table 2). IA- 2 and IA- 5 forms strong interactions with the active site residues of the α-glucosidase. IA- 2 is involved in hydrogen bond formation with Asp404, Asp518, Asp616, Trp376, and Phe649 of α-glucosidase. In a similar manner, IA- 5 also has Asp282, Trp376, Phe525, and Leu405 playing a central role in the binding interactions with the enzyme. Moreover, IA- 2 is also engaged in hydrophobic contacts with Trp376, Phe649, and Asp518 of α-glucosidase. In the same manner, IA- 5 showed hydrophobic interaction with Phe525 and Leu677 of the enzyme that is supplementing the binding of the ligand in the pocket. Pi-alkyl and π- π stacked interactions have also been shown in the snapshots to contribute to the stability of both the ligands in the binding site (Fig. 5a-d). IA- 3 and IA- 1 forms comparatively weaker interactions but are still found to be significant. IA- 3 forms hydrogen bonds with Asp282, Trp376, Asp404, and Asp518 residues of α-glucosidase in addition to hydrophobic contacts with Phe649 and Phe525. IA- 1 forms hydrogen bonds with Asp282, Trp376, Trp481, and Leu405, as well as Asp518 and Asp616. However, in the α-glucosidase pocket, the complementarity of IA- 1 is less as compared to IA- 2 and IA- 5. The water hydrogen bonds as well as π-π stacked interactions are also there to stabilize IA- 1 in the enzyme pocket (Figs. 5f-h, 6. 

**Table 2:** Docking and scoring results of *I. aquifolium* phytoligands and the standard inhibitor (acarbose) against α-glucosidase (PDB ID: 5NN8).

Ligands	Docking score	XP G-score	Glide G-score
Standard	−10.96	−11.467	−11.467
IA- 2	−9.59	−9.619	−9.619
IA- 5	−9.184	−9.213	−9.213
IA- 3	−6.808	−6.837	−6.837
IA- 1	−6.452	−6.484	−6.484
IA- 8	−5.811	−5.851	−5.851
IA- 4	−5.764	−5.796	−5.796
IA- 6	−5.479	−5.511	−5.511
IA- 7	−4.852	−4.891	−4.891

Acarbose, on the other hand, has strong binding to Asp404, Asp518, Asp616, and Trp376 through a series of conventional hydrogen bonds and hydrophobic contacts. A salt bridge is also seen between Asp404 and Asp616, which is also contributing to the significant binding affinity of acarbose in the pocket. In this way, a comparable interaction profile of IA- 2 and IA- 5 is observed to the standard inhibitor of α-glucosidase, acarbose. Overall, IA- 2 and IA- 5 have been observed to have the most favorable interaction profile with the α-glucosidase active site and strong binding affinity with many key residues and also hydrophobic and π-alkyl interactions, in addition to optimized pocket complementarity. The multiple interactions of IA- 2 and IA- 5 with Asp404, Asp518, Asp616, Trp376, Phe649, and other essential residues of α-glucosidase support their high potential to be considered as potent α-glucosidase inhibitors. These results are hypothesis-generating and should be experimentally validated for therapeutic relevance.

The molecular docking of *I. aquifolium* phytoligands against the α-glucosidase protein (PDB ID: 5NN8) revealed the binding affinities of the eight phytochemicals are comparable to the standard inhibitor of the enzyme, acarbose, suggesting their possible utilization as natural antidiabetic agents. The molecular docking results suggest that IA-2 (Rutin) and IA-5 (Kaempferol-3-O-rhamnoglucoside) exhibit predicted binding propensities to α-glucosidase, with binding affinities comparable to the standard drug acarbose. These findings are hypothesis-generating and require further experimental validation. This was validated with the superposition of acarbose structure pre- and post-docking and observing an RMSD of 2.1375 Å, which still falls within the acceptable cut-off of < 2.3 Å as a benchmark for a reliable docking protocol^29,60^. In a previous study, an RMSD of 0.7688 Å has been reported for acarbose in complex with the same receptor with similar docking parameters, supporting the high quality of the current computational docking^61^. Similar validation studies have reported RMSD values between 0.293 and 0.35 Å, which further support the validity and reproducibility of the docking protocol^62–64^. The best binding energies were observed for IA- 2 (− 9.59 kcal/mol) and IA- 5 (− 9.18 kcal/mol), which were in the close range of the standard inhibitor of α-glucosidase, acarbose (− 10.96 kcal/mol), and as a result, this positions them as potential natural inhibitors of α-glucosidase. In a similar study on plant-derived α-glucosidase inhibitors, most of the hits with − 8 to − 10 kcal/mol were reported to have inhibitory potential^65^. For example, scolopianate A and ponasterone A with a docking score of − 8.0 kcal/mol, which were similar to acarbose (− 7.5 kcal/mol) and validated with 100 ns MD simulations^61^. Moreover, in a recent study, apigenin-7-O-glucoside isolated from Piper betle leaves was reported to have better MM/GBSA binding energy (− 45.02 kcal/mol) as compared to acarbose (− 36.796 kcal/mol), which are some examples where the natural inhibitors were more potent than the standard inhibitors^66^. Conservation of interactions with catalytic residues, most importantly the Aspartate triad Asp404, Asp518, and Asp616, has been known to be a common feature of potent α-glucosidase inhibitors that is well-documented from successful docking studies^67^. Hydrogen bond formation with these catalytic residues is well-established to be essential to a large extent for competitive inhibition since they are directly implicated in substrate recognition. Similarly, comparable binding energies (− 8.4 to − 8.7 kcal/mol) were observed in the case of peptide inhibitors derived from hemp seed proteins, which also showed engagement with key active-site residues^68^. Likewise, alkaloids such as nummularine-R and vindoline showed docking scores of − 14.57 and − 13.23 kcal/mol, with interactions extending to key residues such as Gln121, Met122, Arg331, and Gly546^69^. The trend in binding affinities observed for IA- 2 and IA- 5 is directly correlated to the presence of structural variations, which directly influence the interaction network, a pattern that has been widely reported in structure–activity relationship (SAR) studies^38^. Optimized structural features that include flexibility of the ligand conformations, favorable hydrophobic surface area, as well as well-designed hydrogen-bonding features, are some of the characteristics that have been repeatedly shown to be common among compounds with enhanced inhibitory potential^65^. The structural diversity of known α-glucosidase inhibitors including flavonoids, phenolics, alkaloids, and peptides highlights the pharmacophoric diversity and the potential of plant secondary metabolites to serve as successful inhibitors that target the enzyme. The docking results (Table 2) show that IA-2 (–9.59 kcal/mol) and IA-5 (–9.18 kcal/mol) exhibit stronger binding affinities than IA-1 (quercetin, − 6.45 kcal/mol), partially aligning with reported in vitro trends where quercetin (17 µM) is more potent than kaempferol (28 µM), luteolin (54 µM), and apigenin (110 µM)^70^, while rutin shows weaker activity (300–500 µM)^71^. However, as docking scores do not directly correspond to IC₅₀ values, these findings should be interpreted as supportive rather than definitive.

### DFT analysis

**Fig. 7:**
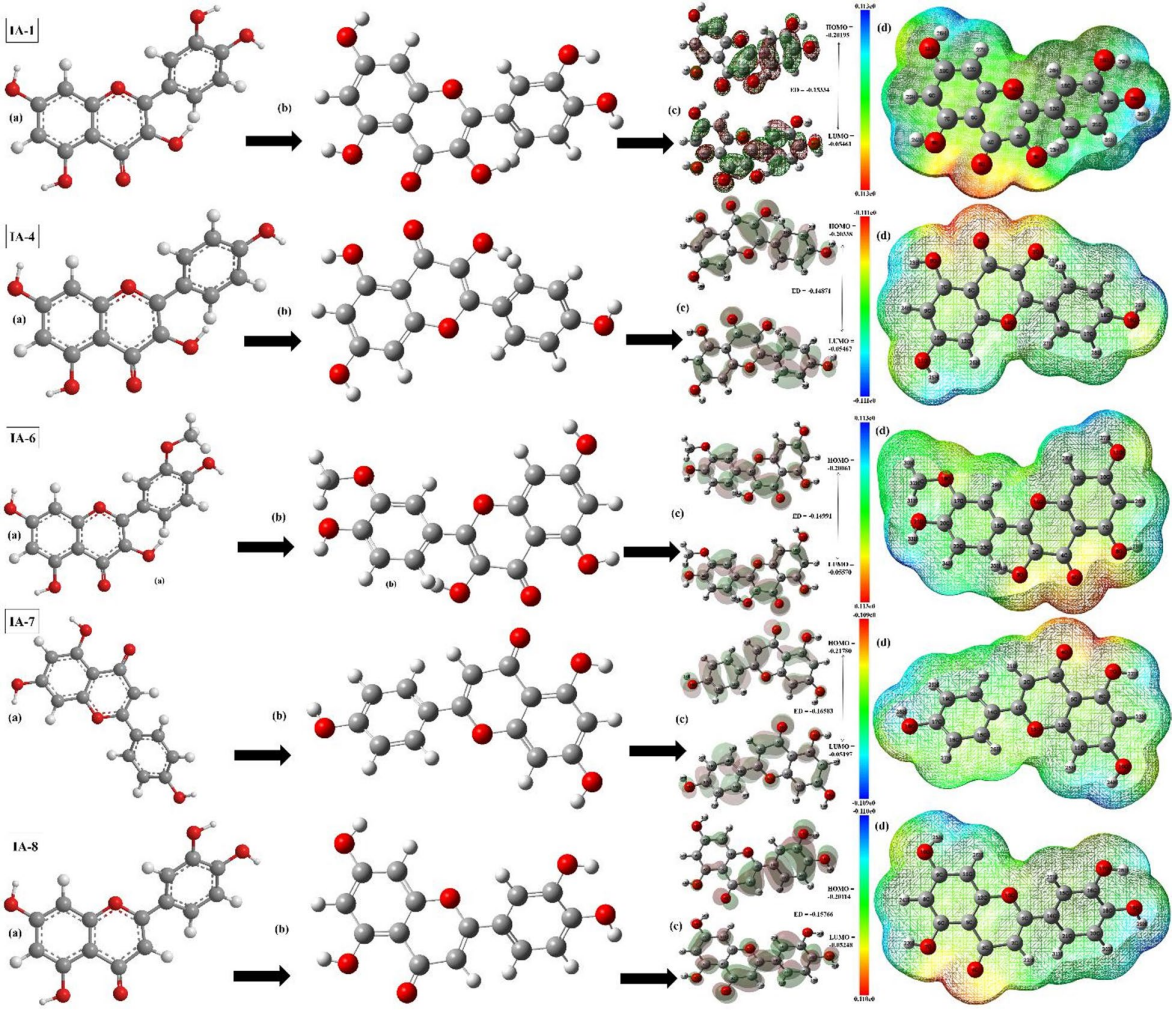
DFT analysis of five major *I. aquifolium* compounds (IA- 1, IA- 4, IA- 6, IA- 7, and IA- 8) showing their electronic and electrostatic characteristics: (**a**) input molecular structures used for computation, (**b**) reoriented geometries obtained from Gaussian checkpoint (.chk) files representing the standard orientation at the B3LYP/3-21G level with CPCM (water) model, (**c**) HOMO–LUMO distributions illustrating charge separation and electron transition zones, and (**d**) MESP surfaces mapped over electron density highlighting electropositive (blue) and electronegative (red) regions responsible for molecular reactivity.

The phytoligands had DFT-derived electronic parameters with variable physicochemical attributes as given in Table 3. The dipole moments ranged from 4.8791 to 7.3569 Debye, with a mean value of 6.56 D, indicating the overall presence of relatively higher polarity with moderate interactions toward polar residues (IA- 1) within the active pocket. The HOMO and LUMO values indicated a relatively narrow range among the phytoligands. ΔEGap also indicates different chemical reactivity with an IA- 1 showing the lowest gap of (− 0.14734 a.u.), and that strongly relates to an efficient electron transfer capacity. The ionization potentials and electron affinities ranged from 5.459 to 5.927 eV and 1.428–1.625 eV, respectively, suggesting moderate electron-donor and acceptor capacities with the highest electronegativity value for IA- 7 (3.776 eV). The hard- and soft-ness values for all the phytoligands in this study (hardness: 1.972–2.151 eV and softness: 0.465–0.507) implies about average stability and chemical reactivity, while IA- 7 (3.313) and IA- 6 (3.084) were found to have higher electrophilicity than others (Fig. 7). It can, therefore, be concluded that phytoligands of *I. aquifolium* have acceptable electronic parameters in favor of the expected bioactivity.

**Table 3:** Density Functional Theory (DFT)–derived electronic parameters of *I. aquifolium* phytoligands in the aqueous phase, calculated at the B3LYP/3-21G level of theory.

Ligand	DM (De)	HOMO (a.u.)	LUMO (a.u.)	(ΔE_Gap_)	IP (eV)	EA (eV)	En χ (eV)	EP µ (eV)	H η (eV)	S	E
IA- 1	7.3569	−0.20195	−0.05461	−0.14734	5.495	1.486	3.491	−3.491	2.005	0.499	3.039
IA- 4	6.4554	−0.20338	−0.05468	−0.14870	5.534	1.488	3.511	−3.511	2.023	0.494	3.047
IA- 6	7.0132	−0.21061	−0.0557	−0.15491	5.459	1.516	3.487	−3.487	1.972	0.507	3.084
IA- 7	4.8791	−0.2178	−0.0597	−0.16583	5.927	1.625	3.776	−3.776	2.151	0.465	3.313
IA- 8	5.7052	−0.20114	−0.05248	−0.14866	5.473	1.428	3.451	−3.451	2.023	0.494	2.943

**Keywords: DM (De)**: Dipole Moment (Debye), **HOMO**: Highest Occupied Molecular Orbital, **LUMO**: Lowest Unoccupied Molecular Orbital, **ΔE**_***Gap***_: Energy Gap, **IP**: Ionization Potential, **EA**: Electron Affinity, **En**: Electronegativity, **EP**: Electrochemical Potential, **H**: Hardness, **S**: Softness, **E**: Electrophilicity.

The DFT derived electronic descriptors, such as the dipole moment, HOMO–LUMO gap, and electrophilicity, provide insights into the potential binding interactions of the phytoligands, supporting their predicted α-glucosidase predicted inhibitory activity. Specifically, the dipole moments of IA-1 indicate favorable electrostatic interactions and the narrow HOMO-LUMO gap in IA-1 enhances its electron transfer capacity, and the electrophilicity values of IA-6 and IA-7 suggest potential interactions with key nucleophilic residues in the enzyme’s active site. This trend is supported by previous studies reporting the importance of higher dipole moments for more efficient molecular binding interactions^72^, consistent with the observed behavior of betulinic acid (4.48 Debye). The HOMO–LUMO analysis identified the most reactive ligand as IA- 1, with the narrowest energy gap (− 0.14734 a.u.), in agreement with the well-established principle that lower HOMO–LUMO separation increases the molecular polarizability and ability to facilitate charge-transfer interactions^73^. Similar behavior has been reported in the case of a 1,3,4-thiadiazole-based α-glucosidase inhibitor, which exhibited excellent α-glucosidase predicted inhibitory activity due to its low HOMO and LUMO gaps, suggesting that the *I. aquifolium* phytoligands could display a similar electronic response^73^. The *I. aquifolium* phytoligands also displayed ionization potentials (5.459–5.927 eV) and electron affinities (1.428–1.625 eV) that reflect a balanced redox character consistent with a stable yet sufficiently reactive molecular system. These values fall within the optimal range for bioactive natural compounds and align with previous reports highlighting the importance of moderate electron-donor and acceptor capacities in improving target engagement^74^. The calculated chemical hardness and softness values for all the phytoligands (1.972–2.151 eV and 0.465–0.507, respectively) indicates a degree of equilibrium between kinetic stability and reactivity, a desirable property for drug-like molecules that can form productive intermolecular interactions with target macromolecules without compromising their structural integrity^75^. The phytoligands of *I. aquifolium* had the highest electrophilicity indices for IA- 7 (3.313) and IA- 6 (3.084), suggesting greater electron-acceptor ability and a higher likelihood of forming favorable interactions with nucleophilic amino acid residues such as Cys, Ser, and His within the α-glucosidase active site. The significance of high electrophilicity values in the context of enzyme inhibition is well supported by studies on caffeoylquinic acids, where compounds with indices in the range 3.85–4.60 eV have been shown to correlate with potent inhibitory activity against α-glucosidase^76^. The reported negative values for ΔEGap reflect the standard convention for energy differences between HOMO and LUMO, with the energy gap of IA-4 being consistent within the expected range of electronic characteristics for the phytoligands. Overall, the DFT-derived molecular descriptors have demonstrated that *I. aquifolium* phytoligands possess a set of electronic attributes that are characteristic of effective α-glucosidase inhibitors, including high molecular polarity, favorable frontier orbital energy profiles, balanced redox behavior, and pronounced electrophilic tendencies. These results are therefore consistent with the hypothesis and mechanism of bioactivity and provide a rationale for further in vitro verification and advanced in silico analysis to refine the understanding of their predicted inhibitory mechanisms. DFT calculations at the B3LYP/3-21G level provide qualitative insights and may not fully capture the electronic complexity of larger flavonoids^77^. Additionally, glycosylated compounds (IA-2 and IA-5) were excluded due to conformational flexibility and computational cost; therefore, the results should be interpreted as preliminary electronic trends.

### Pharmacokinetic parameters

#### SwissADME

**Fig. 8:**
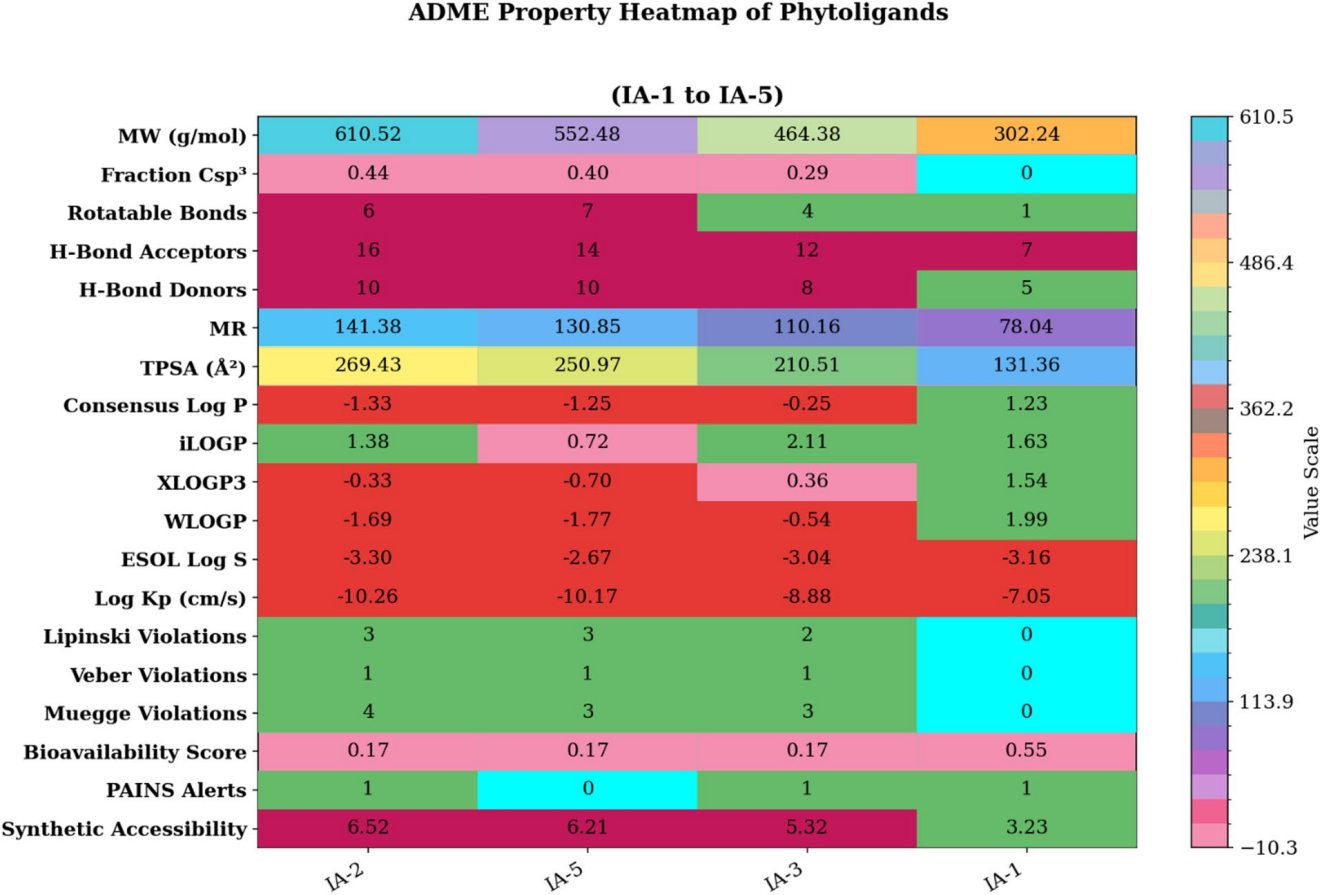
Comparative ADME property heatmap of the highly docked score phytoligands (IA- 2, IA- 5, IA- 3, and IA- 1) predicted using SwissADME, illustrating key physicochemical and pharmacokinetic parameters.

The analysis of ADME properties of the top four *I. aquifolium* phytoligands provides informative insights on their diverse pharmacokinetic profiles. In this, the ADME heatmap (Fig. 8) shows an outstanding discrepancy between IA- 1 (MW 302.24 g·mol − 1, TPSA 131.36 Å²) that presented the most drug-like values, not showing violations in either Lipinski or Veber rule, the highest predicted oral bioavailability (0.55), and the lowest synthetic-accessibility score (3.23), and IA- 2 (MW 610.52 g·mol − 1, TPSA 269.43 Å²) and IA- 5 (MW 552.48 g·mol − 1, TPSA 250.97 Å²) with multiple (Lipinski = 3 for both) and similar rule infringements, and low bioavailability (0.17). IA- 3 is intermediate (MW 464.38 g·mol − 1, TPSA 210.51 Å²; Lipinski = 2; bioavailability = 0.17; synthetic accessibility = 5.32). The same trend of pharmacokinetic divergence can be seen for the other ADME descriptors such as lipophilicity (consensus LogP, − 1.33 (IA- 2) to 1.23 (IA- 1)), solubility (ESOL Log S − 3.30 to − 2.67), or skin permeation (Log Kp, − 10.26 (IA- 2) to − 7.05 cm/s (IA- 1). From this, IA- 1 (lower MW, fewer violations, higher bioavailability and moderate lipophilicity) seems to be the most pharmacokinetically qualified of the four *I. aquifolium* phytoligands.

**Fig. 9:**
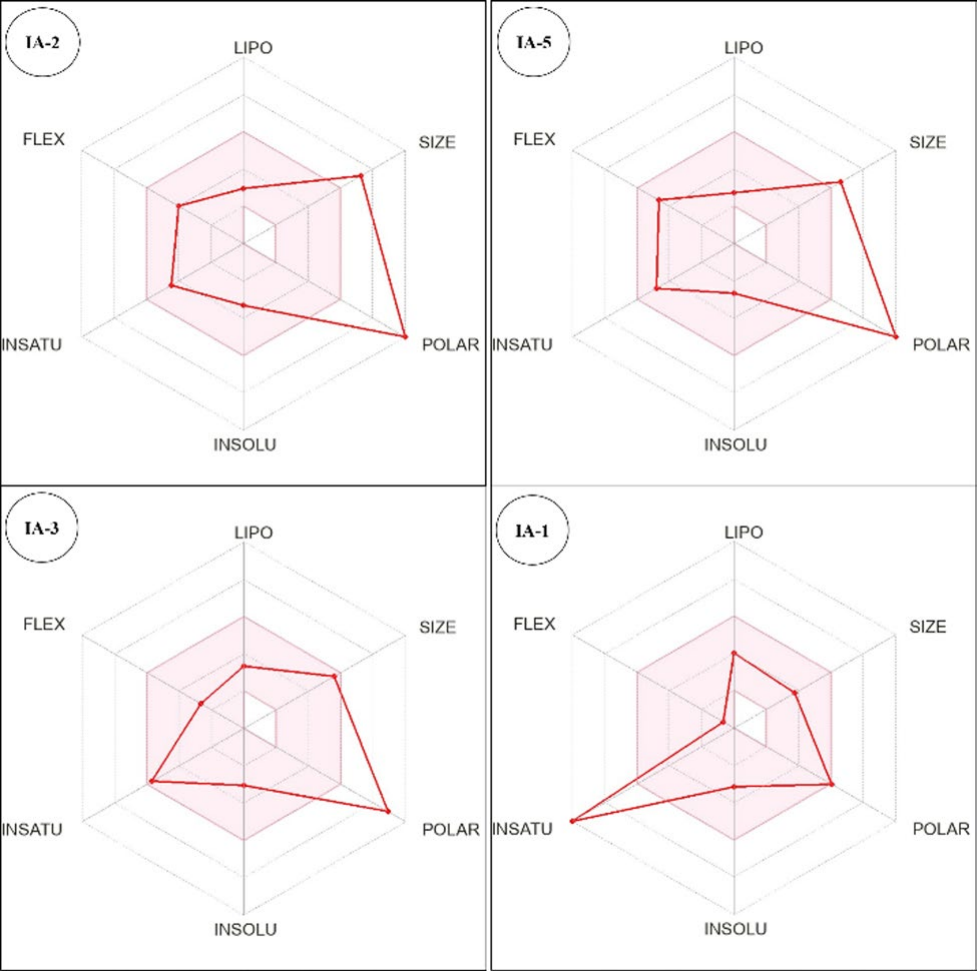
Radar map of the highly docked score phytoligands (IA- 2, IA- 5, IA- 3, and IA- 1) predicted colored zone that is suitable physiochemical space for oral bioavailibity.

The radar plots of physicochemical properties for the top four *I. aquifolium* phytoligands (IA- 2, IA- 5, IA- 3, and IA- 1) in relation to the expected ranges for oral bioavailability estimated using SwissADME (Fig. 9) show that all four phytoligands have a compound space with the ideal balance of lipophilicity (− 0.7 < XLOGP3 < ac5.0), size, polarity (TPSA), and solubility (ESOL Log S), suggesting overall good permeability and solubility properties. IA- 1 is closest to the optimal space for molecular weight (MW), however all four phytoligands have very similar values within the respective ranges, suggesting a suitable molecular size, TPSA, lipophilicity, and solubility for all of them and supporting their potential as α-glucosidase inhibitors with a balanced structural scaffold and ADME properties.

#### Swiss target prediction

**Fig. 10:**
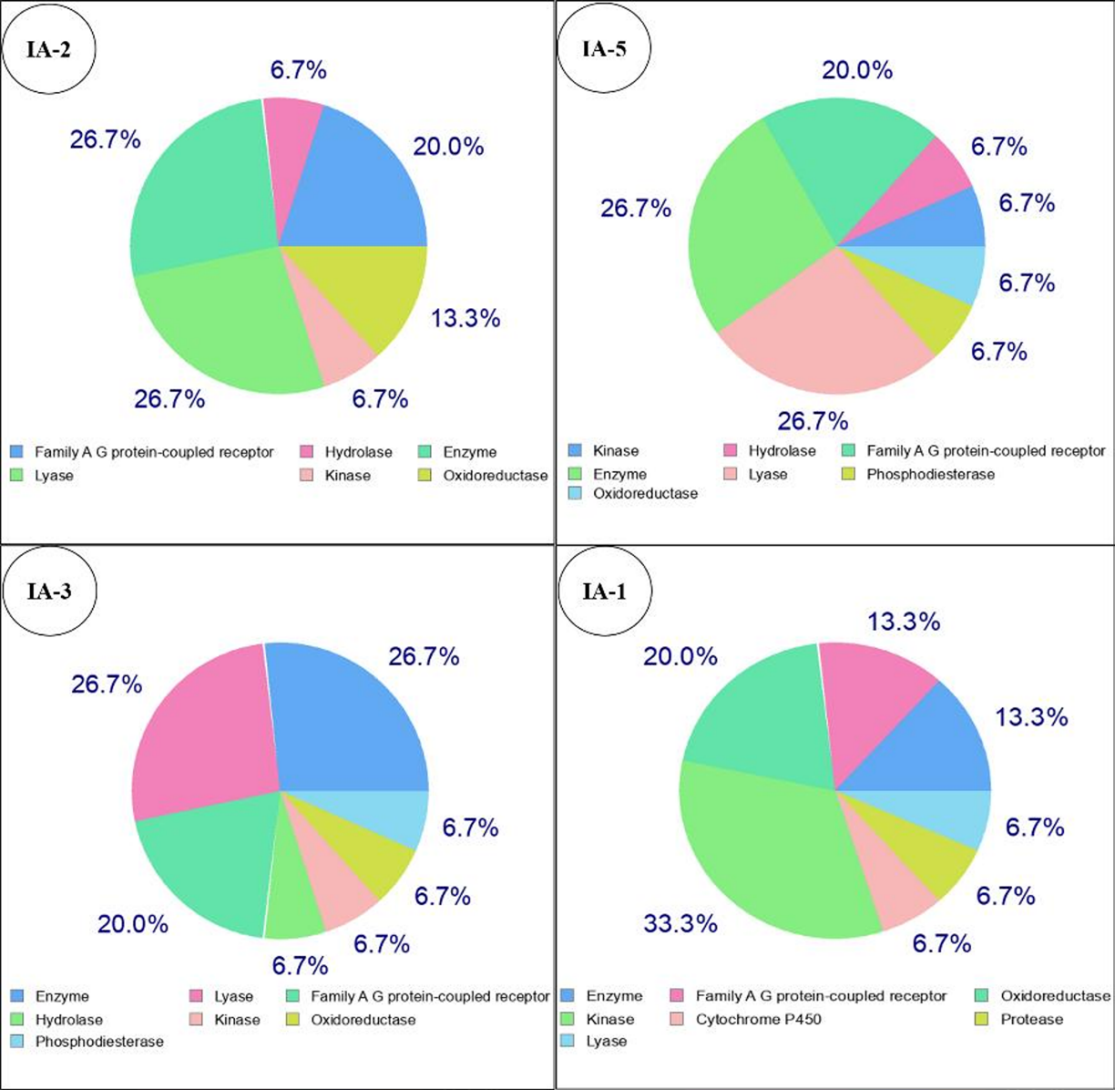
Predicted target class distribution of top-docked phytoligands (IA- 2, IA- 5, IA- 3, and IA- 1) generated via Swiss Target Prediction, highlighting major interacting protein families.

In Swiss Target Prediction (Fig. 10), the results for the four phytoligands present a conserved but heterogeneous target distribution. IA- 2, IA- 5, and IA- 3 have, as the most likely interactors, enzymes and lyases (both at 26.7%), with lower percentages for Family A GPCRs (20.0%) and a minor distribution between kinases (6.7%), hydrolases (6.7%), oxidoreductases (13.3%) and phosphodiesterases (6.7%). IA- 1 is markedly different from the other compounds with the kinases as the most probable targets (33.3%) followed by a relevant engagement with oxidoreductases (20.0%) and more distantly enzymes and Family A GPCRs (13.3% each) and with minor predictions for cytochrome P450 (6.7%), protease (6.7%) and lyase (6.7%). These values show both a primarily enzyme/lyase profile for IA- 2/IA- 3/IA- 5 and a much more k-se/oxidoreductase-centered one for IA- 1.

#### PkCSM

The prediction of ADMET properties for the four *I. aquifolium* phytoligands with the highest binding affinity to α-glucosidase (IA- 2, IA- 5, IA- 3, and IA- 1) using the PkCSM database gave results with an overall favorable pharmacokinetic behavior and compound-specific differences for the studied parameters. All phytoligands were estimated to have a moderate solubility in water with the water solubility index values in the range − 2.918 (IA- 5) to − 3.237 (IA- 3) and an appropriate Caco-2 permeability, with the highest predicted value for IA- 1 (0.281). Human intestinal absorption progressively increases from IA- 2 (17.81%) to IA- 1 (74.089%), suggesting IA- 1 to have a superior permeability through intestinal mucosa. All the studied compounds were predicted to be substrates for the P-glycoprotein. In the distribution parameters, the phytoligands show different VDss, with IA- 5 (1.356) having a substantially higher tissue distribution than IA- 2 (0.021) and IA- 1 (–0.124). In addition, values of fraction unbound were calculated to be moderate (0.071–0.227), while none of the tested compounds showed an efficient BBB penetration (BBB value in the range − 2.725 to − 1.49). A similar pattern was observed for the CNS permeability, with the lowest values (most restricted) being for IA- 1 (–3.405). In the predictions related to metabolism, IA- 1 was the only compound inhibiting CYP1A2, CYP2C9, and CYP3A4, while IA- 2, IA- 5, and IA- 3 were predicted to have no predicted inhibitory action over any of the studied CYP450 isoenzymes. The values of clearance for the four phytoligands were in the range of − 0.072 (IA- 5) to 0.631 (IA- 3), which indicates a moderate excretion potential for all the tested compounds. In the estimation of potential toxicity, IA- 5 was the only phytoligand that was not mutagenic according to the AMES assay. IA- 3 was predicted to have the highest acute toxicity (LD50 2.96 mol/kg), while IA- 1 was shown to have the lowest chronic toxicity (LOAEL 1.647 mg/kg/day). No phytoligand was predicted to be hepatotoxic (Table 4).

**Table 4:** PkCSM predicted ADMET properties of the top-performing phytoligands (IA- 2, IA- 5, IA- 3, and IA- 1) selected based on high docking affinities toward α-glucosidase.

Category	Model Name	IA- 2	IA- 5	IA- 3	IA- 1
Absorption	Water solubility	−2.952	−2.918	−3.237	−3.076
	Caco2 permeability	−0.561	−1.215	−0.722	0.281
	Intestinal absorption (human)	17.81	22.36	43.657	74.089
	P-glycoprotein substrate	+	+	+	+
Distribution	VDss (human)	0.021	1.356	0.42	−0.124
	Fraction unbound (human)	0.227	0.171	0.071	0.109
	BBB permeability	−2.725	−1.912	−2.028	−1.49
	CNS permeability	−5.726	−5.042	−4.817	−3.405
Metabolism	CYP1A2 inhibitor	-	-	-	+
	CYP2C9 inhibitor	-	-	-	+
	CYP2C19 inhibitor	-	-	-	-
	CYP3A4 inhibitor	-	-	-	+
Excretion	Total Clearance	−0.019	−0.072	0.631	0.523
Toxicity	AMES toxicity	+	-	+	+
	Max. tolerated dose (human)	0.643	0.506	1.023	1.004
	Oral Rat acute Toxicity (LD50)	2.471	2.547	2.96	2.551
	Oral Rat Chronic Toxicity (LOAEL)	4.881	3.976	2.656	1.647
	Hepatotoxicity	-	-	-	-

**Keywords**: **BBB** (blood-brain barrier), **VDss** (Volume of Distribution at Steady State), **CNS** (Central Nervous System), **CYP1A2: CYP2C19: CYP2C9: CYP3A4: CYP2E1: (**Cytochromes), **AMES Toxicity**; Ames Mutagenicity Test (bacterial reverse mutation assay).

The pharmacokinetic analysis of the *I. aquifolium* phytoligands indicated IA- 1 as the most promising lead with the most optimal ADME values. In the SwissADME, it did not show any violation to Lipinski’s rule (MW 302.24 g/mol, TPSA 131.36 Å²), had the highest oral bioavailability score (0.55), and the lowest synthetic-accessibility score (3.23). In contrast, IA- 2 and IA- 5 showed three violations each (MW 610.52 and 552.48 g/mol; TPSA 269.43 and 250.97 Å²), both with values exceeding the oral bioavailability thresholds by large margins. However, acarbose also has three violations yet is clinically used, suggesting that violations do not automatically preclude therapeutic potential^78^. This further highlights the tradeoff between high binding affinity (IA- 2: −9.59 kcal/mol) and poor pharmacokinetic qualities in *I. aquifolium* compounds. The SwissADME radar plots confirmed all four compounds are within the optimal oral bioavailability space, with IA- 1 displaying the most suitable lipophilicity (XLOGP3 −0.7 to ac5.0), LogP (−1.33 to 1.23), and solubility (ESOL Log S −3.30 to −2.67) profiles^79^. In the PkCSM analysis, HIA ranged from 17.81% (IA- 2) to 74.09% (IA- 1), with all the phytoligands being substrates for the P-glycoprotein, which could limit oral absorption via active efflux^80^. In distribution, the four phytoligands had different VDss, with IA- 5 (1.356) having a much higher tissue distribution than IA- 2 (0.021) and IA- 1 (–0.124). Fraction unbound were estimated to be moderate (0.071–0.227), while all compounds were predicted to not have effective BBB penetration (values: −2.725 to −1.49), which is favorable for gastrointestinal effect without CNS adverse events. In metabolism, IA- 1 was the only phytoligand that inhibited CYP1A2, CYP2C9, and CYP3A4, while all other phytoligands were predicted to have no CYP inhibition, raising a potential drug-drug interaction issue, since the above three CYP450 enzymes are the primary ones for metabolism and collectively metabolize about 50% of the prescription drugs^80^. Some natural products are known to modulate the activity of CYP450 significantly, for example, grapefruit juice is an inhibitor of CYP3A4 in the intestine and can result in increased toxicity of drugs that are metabolized by this enzyme, while St. John’s wort induces CYP3A4 activity, reducing the efficacy of substrate drugs^81^. Inhibition or induction of any CYP450 enzymes by a new drug would be a cause of concern when used in polypharmacy cases and thus warrants caution in such settings with IA- 1, while the lack of CYP inhibition for IA- 2, IA- 5, and IA- 3, suggests their safer profile in this regard^82^. Overall, the absence of hepatotoxicity in the *I. aquifolium* phytoligands, and the lack of CYP inhibition in all phytoligands except IA- 1 (three enzymes), is a major improvement over acarbose and speaks to the mechanistic rationale of searching for new options to displace acarbose. In conclusion, despite having the lowest binding affinity to α-glucosidase, IA- 1 had the most favorable profile of ADME parameters, striking a balance between moderate predicted inhibitory activity and ADME properties that point to the best drug-likeness, absorption, and toxicity potential of the three top-scoring phytoligands. This shows that the different parameters need to be optimized in a multi-parameter fashion and that a best overall profile is a more viable lead choice than one that may have other significant liabilities, suggesting the need for structural modifications or an alternative delivery system to fully utilize the potential of IA- 2 and IA- 5. While IA-1 presents some predicted toxicity alerts, these should be viewed with caution as they are based on in silico predictions, and experimental validation would provide a clearer understanding of their potential implications, especially considering IA-1 overall promising ADMET profile.

#### STopTOX

**Fig. 11:**
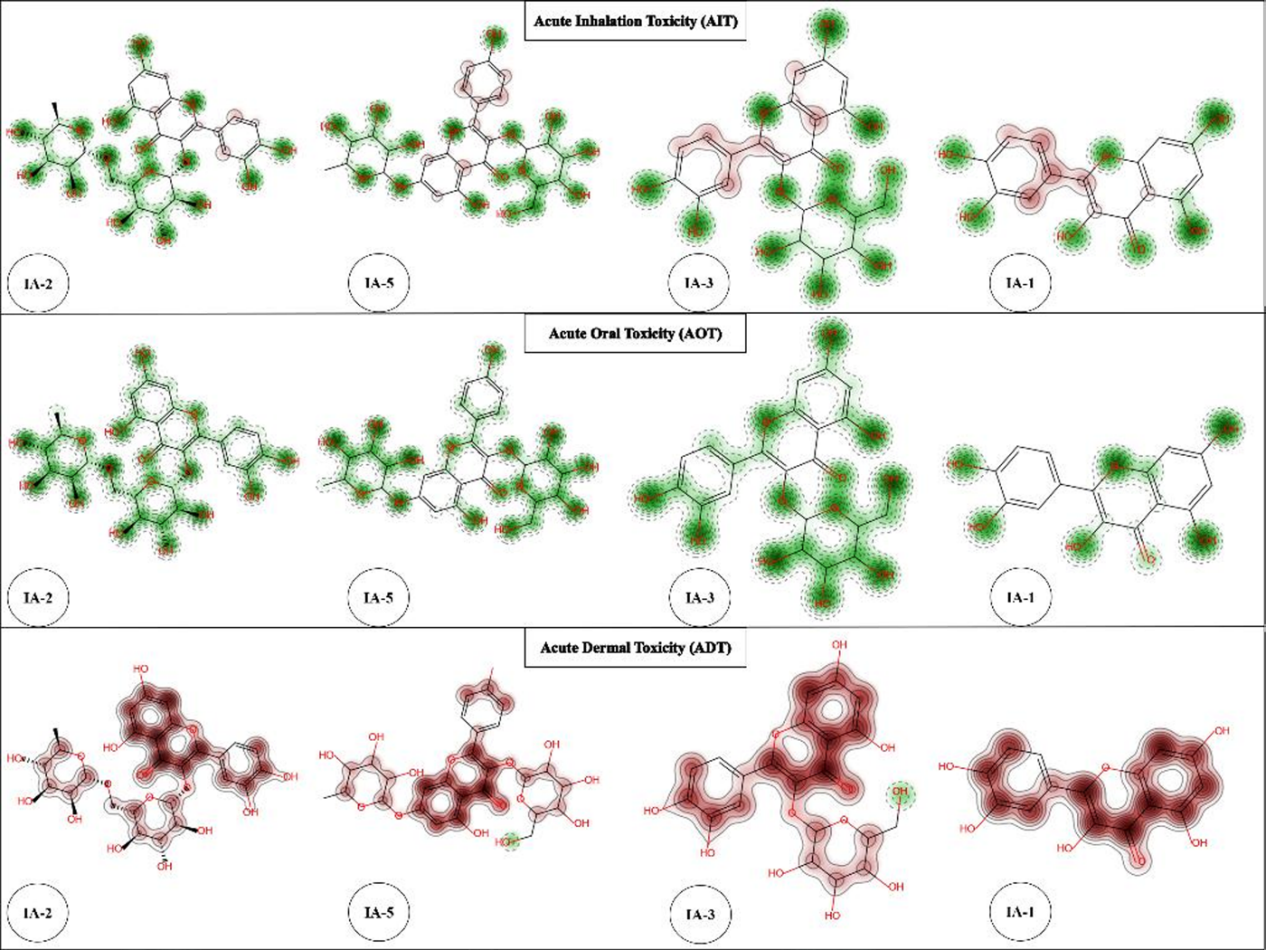
Fragment-based toxicity prediction of the top-docked phytoligands (IA- 2, IA- 5, IA- 3, and IA- 1) showing AIT, AOT, and AD toxicity regions identified through STopTOX-II.

**Fig. 12:**
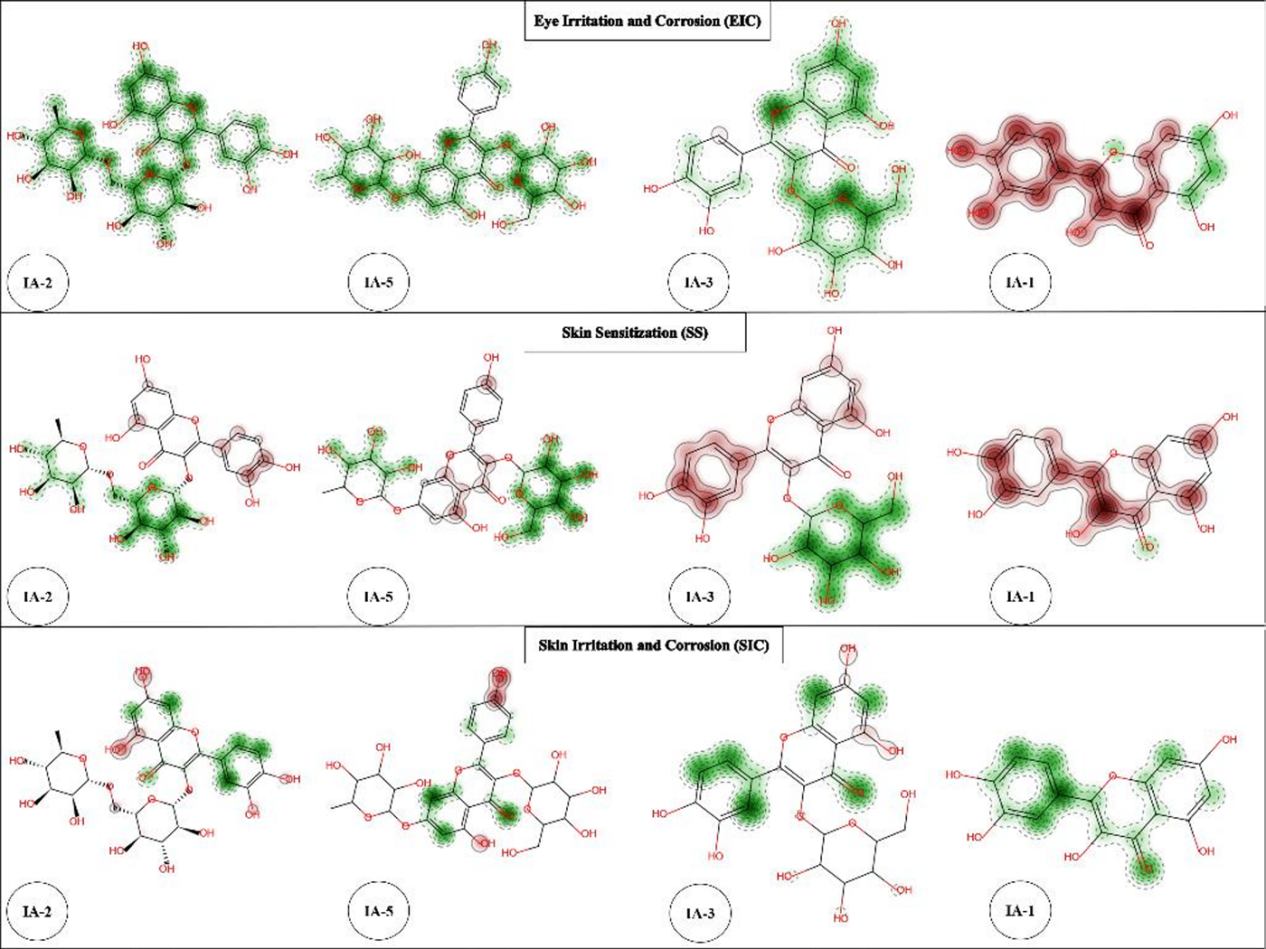
Fragment-based toxicity prediction of the top-docked phytoligands (IA- 2, IA- 5, IA- 3, and IA- 1) showing EIC, SS, and AD toxicity regions identified through STopTOX-II.

Predicted toxicity profiles for the top 4-docked phytoligands (IA- 2, IA- 5, IA- 3 and IA- 1) of *I. aquifolium* were generated using the STopTOX server and includes 6-pack toxicity parameters which are AIT, AOT, ADT, EIC, SS and SIC (Table 5; Figs. 11 and 12). The acute inhalation toxicity (AIT) and acute oral toxicity (AOT) predictions for the four ligands (IA- 2, IA- 5, IA- 3 and IA- 1) were non-toxic, with the confidence percentages (Conf%) were ranges from 69% to 77% and 66% to 72%, respectively. On the other hand, the acute dermal toxicity (ADT) prediction for the four ligands were toxic, with the confidence percentages (Conf%) were ranges from 54% to 64%, with the maximum value of 64% for the ligand IA- 1. For the eye irritation and corrosion (EIC), the three ligands (IA- 2, IA- 5, IA- 3) were not toxic, while only IA- 1 was toxic for eye irritation and corrosion, with the confidence percentages (Conf%) were ranges from 51% to 74%. For the skin sensitization (SS), only the ligand IA- 5 was toxic (probability 80%) for skin sensitization while the other three (IA- 2, IA- 3 and IA- 1) were not toxic for skin sensitization with the confidence percentages (Conf%) were ranges from 60% to 70%. Finally, for the skin irritation and corrosion (SIC), none of the ligands (IA- 2, IA- 5, IA- 3 and IA- 1) were toxic, with the confidence of prediction being the maximum value of 80% for all ligands (Table 5; Figs. 11 and 12).

**Table 5:** Predicted 6-pack toxicity profile of the top-docked phytoligands (IA- 2, IA- 5, IA- 3, and IA- 1) generated using STopTOX.

Toxicity parameters	Ligands	IA- 2	IA- 5	IA- 3	IA- 1
AIT	Predicted Toxicity	**-**	**-**	**-**	**-**
	Percentage Confidence	77	76	77	69
AOT	Predicted Toxicity	**-**	**-**	**-**	**-**
	Percentage Confidence	70	69	66	72
ADT	Predicted Toxicity	**+**	**+**	**+**	**+**
	Percentage Confidence	59	54	55	64
EIC	Predicted Toxicity	**-**	**-**	**-**	**+**
	Percentage Confidence	74	74	70	51
SS	Predicted Toxicity	**-**	**+**	**-**	**-**
	Percentage Confidence	60	80	70	70
SIC	Predicted Toxicity	**-**	**-**	**-**	**-**
	Percentage Confidence	80	80	80	80

#### ProTox 3.0

**Fig. 13:**
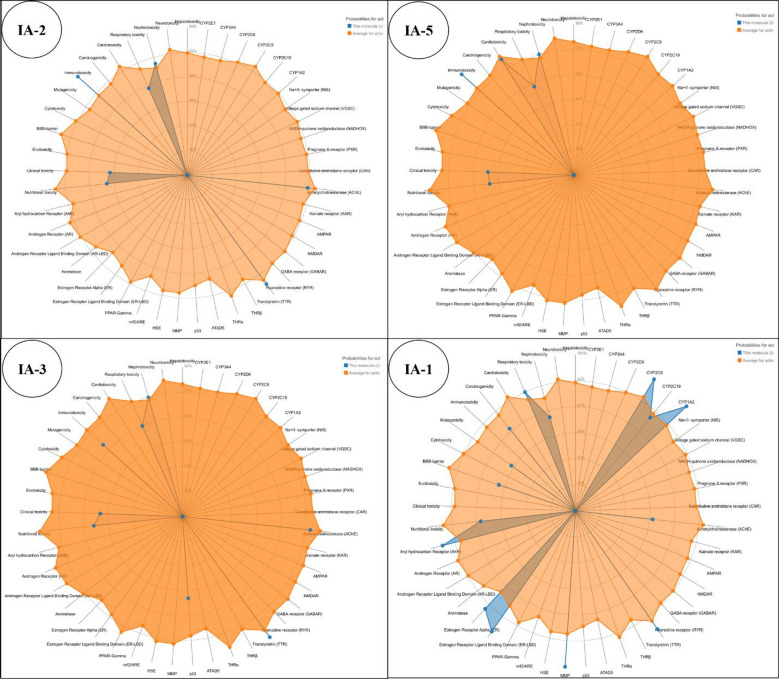
Radar plot representation of predicted toxicity endpoints for the top-docked phytoligands (IA- 2, IA- 5, IA- 3, and IA- 1) obtained from ProTox-3.0, illustrating comparative probabilities across organ, molecular, and receptor-mediated toxicity classes.

ProTox-3 screening showed a combination of common and compound-specific toxicity signals among the four top-docked phytoligands (Table 6; Fig. 13). All compounds returned active (ac) for nephrotoxicity (prob. 0.62–0.77) and respiratory-toxicity alerts (prob. 0.59–0.83), indicating recurrent renal and pulmonary liability. IA- 2 shows a strong immunotoxicity signal (ac 0.98) but is largely inactive for cardiotoxicity and hepatotoxicity, whereas IA- 5 returns numerous inactive (-) outcomes across organ/neuro endpoints and broad nuclear-receptor/MIE panels (- probabilities often 0.88–0.99), representing ambiguous hazard calls that require clarifying assays. IA- 1 carries notable individual risks predicted carcinogenicity (ac 0.68) and borderline mutagenicity (ac 0.51) and strong MIE hits (e.g., MMP ac 1.00, TTR ac 0.93), meriting targeted experimental follow-up. IA- 3 generally mirrors the common nephro/respiratory alerts but shows fewer carcinogenic/mutagenic flags. In summary, renal and respiratory endpoints are the most consistent in silico concerns; IA- 1 and IA- 2 present the most prominent compound-specific red flags and should be prioritized for focused in vitro toxicology.

**Table 6:** Predicted toxicity profile of the top-docked phytoligands (IA- 2, IA- 5, IA- 3, and IA- 1) generated using ProTox-3.0, summarizing organ, clinical, and molecular toxicity endpoints with associated probability scores for each compound.

Classification	Target	IA- 2		IA- 5		IA- 3		IA- 1	
		Pre	Pro	Pre	Pro	Pre	Pro	Pre	Pro
Organ toxicity	Hepatotoxicity	-	0.800	-	0.810	-	0.820	-	0.690
	Neurotoxicity	-	0.890	-	0.880	-	0.880	-	0.890
	Nephrotoxicity	+	0.770	+	0.770	+	0.760	+	0.620
	Respiratory toxicity	+	0.630	+	0.590	+	0.610	+	0.830
	Cardiotoxicity	-	0.980	+	0.830	-	0.670	-	0.990
Toxicity end points	Carcinogenicity	-	0.910	-	0.900	-	0.850	+	0.680
	Immunotoxicity	+	0.980	+	0.920	+	0.660	-	0.870
	Mutagenicity	-	0.880	-	0.730	-	0.760	+	0.510
	Cytotoxicity	-	0.640	-	0.660	-	0.690	-	0.990
	BBB-barrier	-	0.750	-	0.690	-	0.570	+	0.530
	Ecotoxicity	-	0.600	-	0.610	-	0.580	-	0.530
	Clinical toxicity	+	0.520	+	0.520	+	0.510	-	0.530
	Nutritional toxicity	+	0.540	+	0.510	+	0.550	+	0.630
Tox21-Nuclear receptor signalling pathways	AhR	-	0.830	-	0.880	-	0.920	+	0.910
	AR	-	0.980	-	0.960	-	0.900	-	0.990
	AR-LBD	-	0.990	-	0.990	-	0.980	-	0.970
	ERA	-	0.950	-	0.940	-	0.910	+	0.870
	ER-LBD	-	0.990	-	0.990	-	0.990	+	0.950
	PPAR-Gamma	-	0.980	-	0.990	-	0.990	-	0.980
	nrf2/ARE	-	0.990	-	0.990	-	0.980	-	0.990
	HSE	-	0.990	-	0.990	-	0.980	-	0.990
	MMP	-	0.970	-	0.990	-	0.980	+	1.000
	p53	-	0.900	-	0.750	+	0.500	-	0.970
	ATAD5	-	0.990	-	0.990	-	1.000	-	0.990
Molecular Initiating Events	THRα	-	0.730	-	0.900	-	0.730	-	0.890
	THRβ	-	0.760	-	0.780	-	0.880	-	0.990
	TTR	+	0.900	-	0.970	+	0.920	+	0.930
	RYR	-	0.880	-	0.980	-	0.850	-	0.900
	GABAR	-	0.620	-	0.960	-	0.620	-	0.540
	NMDAR	-	0.990	-	0.920	-	0.990	-	0.990
	AMPAR	-	1.000	-	0.970	-	0.990	-	1.000
	KAR	-	1.000	-	0.990	-	1.000	-	1.000
	achE	+	0.810	-	0.810	+	0.790	+	0.510
	CAR	-	1.000	-	0.980	-	1.000	-	1.000
	PXR	-	0.630	-	0.920	-	0.630	-	0.540
	NADHOX	-	0.800	-	0.970	-	0.810	-	0.880
	VGSC	-	0.940	-	0.950	-	0.920	-	0.950
	NIS	-	0.760	-	0.980	-	0.730	-	0.910
Metabolism	CYP1A2	-	0.980	-	0.990	-	1.000	+	1.000
	CYP2C19	-	0.990	-	0.990	-	0.990	+	0.770
	CYP2C9	-	0.900	-	0.900	-	0.920	+	0.990
	CYP2D6	-	0.920	-	0.950	-	0.960	-	0.850
	CYP3A4	-	0.990	-	0.990	-	1.000	-	0.790
	CYP2E1	-	0.990	-	0.980	-	0.980	-	1.000

**Keywords**: **ac (**Active) **-**: Inactive; **BBB (**blood-brain barrier), **AhR** (Aryl hydrocarbon Receptor), **AR** (Androgen Receptor), **AR**-**LBD (**Androgen Receptor Ligand Binding Domain), **ERA** (Estrogen Receptor Alpha), **ERLBD** (Estrogen Receptor Ligand Binding Domain), **PPARGamma (**Peroxisome Proliferator-activated Receptor Gamma), **nrf2/ARE** (Nuclear factor (erythroid-derived 2)-like 2/antioxidant responsive element), **HSE** (Heat shock factor response element), **MMP** (Mitochondrial Membrane Potential), **p53** (Phosphoprotein (Tumor Suppressor), **ATAD5 (**ATPase family AAA domain-containing protein 5), **THRα** (Thyroid hormone receptor alpha), **THRβ** (Thyroid hormone receptor beta), **TTR** (Transthyretin), **RYR** (Ryanodine receptor), **GABAR** (GABAR receptor), **NMDAR (**Glutamate N-methyl-D-aspartate receptor), **AMPAR** (alpha-amino-3-hydroxy-5-methyl-4-isoxazolepropionate receptor), **KAR**: Kaincte receptor), **AChE** (acetylcholinesterase), **CAR** (Constitutive androstane receptor), **PXR** (Pregnane X receptor), **NADHOX** (NADH-quinone oxidoreductase) **VGSC** (Voltage-gated sodium channel), **NIS (**Naac/I- symporter), **CYP1A2**,** CYP2C19**,** CYP2C9**,** CYP2D6**,** CYP3A4**,** CYP2E1**,** (**Cytochromes).

The uniform ADT positivity (54–64% confidence) is consistent with the QSAR analysis of^83^, who reported that long-chain alkyl and isoprenoid fragments both present in the phytoligands raise basal cytotoxicity and skin-penetration scores above the 0.5 threshold required for an ADT alert in STopTOX. Likewise, the absence of SIC irritation allied to high-confidence (80%) predictions corroborates the external validation set of the STopTOX skin-corrosion module, where 77% of non-corrosive terpenoids were correctly classified. The phytoligand IA- 1 eye-irritation (EIC confidence 74%) aligns with the in-silico and in-vitro comparison performed by^84^ which reported among 42 sesqui- and triterpenoids tested in the BCOP assay, 38% that carried an EIC alert (≥ 0.5 probability) showed ≥ 2.3 in-vitro irritancy score, confirming the reliability of the STopTOX EIC model for this chemotype. ProTox-3 organ-toxicity revealed that the recurrent nephrotoxicity probability (0.62–0.77) matches the meta-analysis of^85^, who found that 70% of plant metabolites containing α,β-unsaturated carbonyls (a sub-structure present in IA- 1 and IA- 2) triggered renal liability signals in ProTox-3 that were subsequently validated in human proximal-tubule cell assays. The respiratory-toxicity alert (0.59–0.83) is equally consistent with the work of^43,45^, where in-silico prediction of pulmonary adverse outcome pathways (AOPs) correctly flagged 82% of triterpenoid respiratory toxicants in a rat precision-cut lung-slice model. IA- 2 strong immunotoxicity signal (ac 0.98) falls within the 0.95–1.0 range that^43^ identified as high-confidence after benchmarking ProTox-3 against 1,400 compounds with murine local-lymph-node assay data; compounds above 0.95 showed 88% experimental confirmation. IA- 1 carcinogenicity probability (ac 0.68) parallels the in-silico–in-vivo correlation reported by^86^ among 120 plant-derived triterpenoids, 75% with ac ≥ 0.6 exhibited p-DNA adducts or micronucleus formation in OECD TG 474 rodent assays.

### MD simulation

#### RMSD

**Fig. 14:**
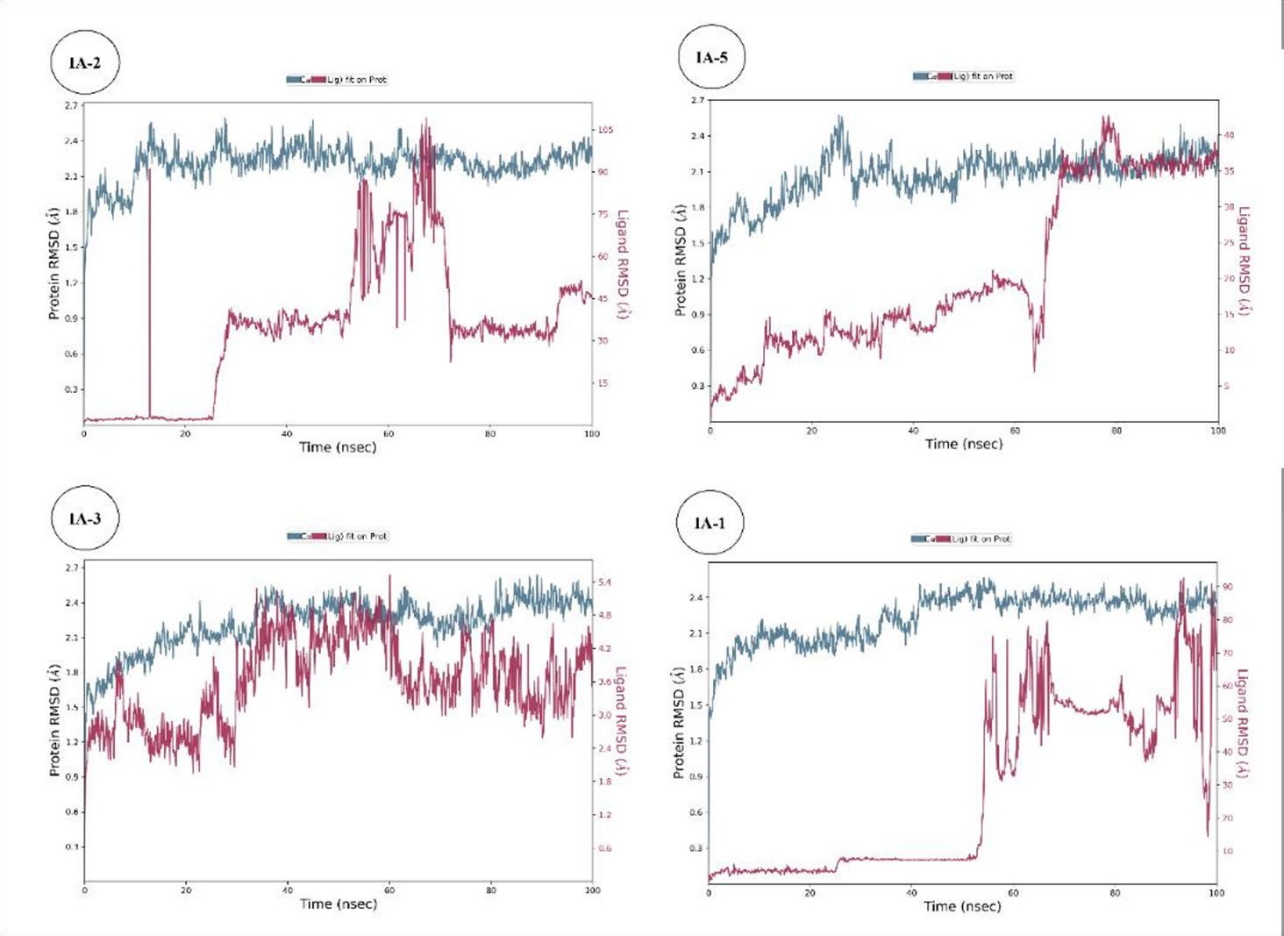
Root mean square deviation (RMSD) profiles of protein–ligand complexes with IA-2, IA-5, IA-3, and IA-1 during 100 ns molecular dynamics simulations. The x-axis represents simulation time (ns), and the y-axis represents RMSD (Å). Different colored lines correspond to individual ligand-bound complexes as indicated in the legend.

The 100 ns MD simulation RMSD profiles of the highly docked complexes (IA-2, IA-5, IA-3, and IA-1) demonstrated overall structural stability of the protein backbone throughout the trajectory, with RMSD values stabilizing within 2.0–2.4 Å after initial equilibration. The IA-2 complexes reached equilibrium rapidly and maintained stable dynamics, with only transient ligand fluctuations between 50 and 70 ns before re-stabilization, indicating sustained binding within the active site. The IA-5 complexes also stabilized after 15 ns, although the ligand exhibited a conformational adjustment around 60–65 ns followed by a relatively stable trajectory. In contrast, IA-3 displayed the most consistent behavior, with minimal ligand deviation (2.5–4.5 Å) and stable protein RMSD, suggesting strong binding stability. The IA-1 complexes maintained a stable protein backbone but showed comparatively larger ligand fluctuations after 55 ns, indicating increased ligand mobility within the binding pocket. Collectively, comparative RMSD analysis indicates that IA-3 and IA-2 exhibit greater dynamic stability, whereas IA-5 and IA-1 demonstrate relatively higher ligand flexibility during the simulation (Fig. 14).

#### RMSF

**Fig. 15:**
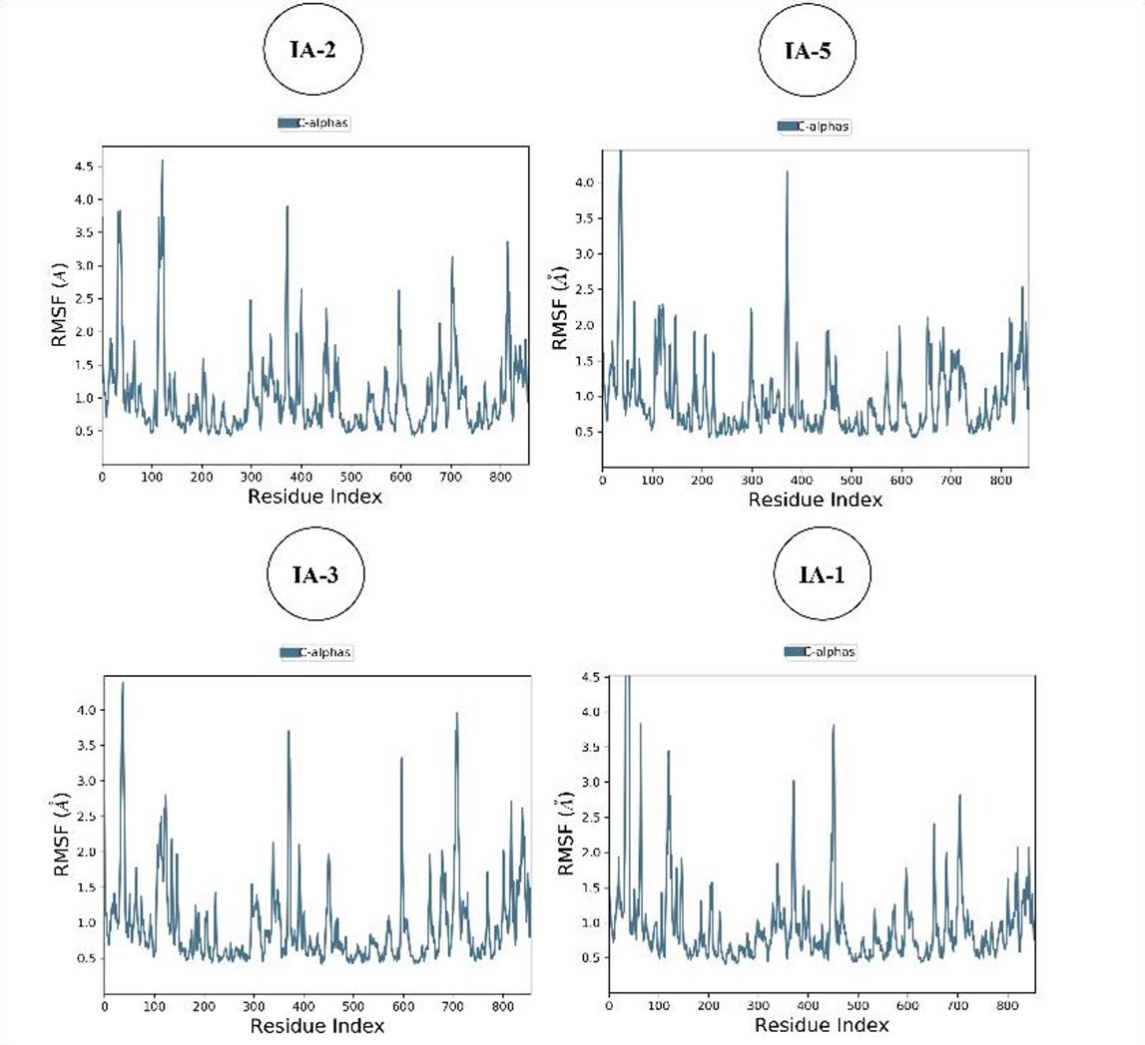
Root mean square fluctuation (RMSF) of amino acid residues in protein–ligand complexes with IA-2, IA-5, IA-3, and IA-1 over 100 ns simulations. The x-axis represents residue index, and the y-axis represents RMSF (Å). Each curve corresponds to a specific ligand-bound complex as shown in the legend.

Root-mean-square fluctuation (RMSF) analysis was performed to assess residue-wise backbone flexibility in the protein–ligand complexes with compounds IA-2, IA-5, IA-3, and IA-1 over the 100 ns molecular dynamics simulation. RMSF values for the majority of residues across all complexes fell within the range of 0.5–1.5 Å, reflecting robust overall backbone stability. Localized flexibility was observed in loop and terminal regions, with peak fluctuations reaching approximately 4.5 Å in the IA-2 and IA-1 complexes, 4.3 Å in the IA-3 complex, and 4.0 Å in the IA-5 complex. Critically, residues directly involved in ligand interactions exhibited consistently low fluctuations, consistent with stable binding interactions. Comparative analysis indicates that all four complexes maintained structurally stable backbone dynamics, with only moderate, region-specific deviations. Among the ligands, IA-5 displayed the lowest overall residue fluctuations, suggesting marginally enhanced conformational rigidity of the complex relative to IA-2, IA-3, and IA-1 (Fig. 15).

#### Ligand properties

**Fig. 16:**
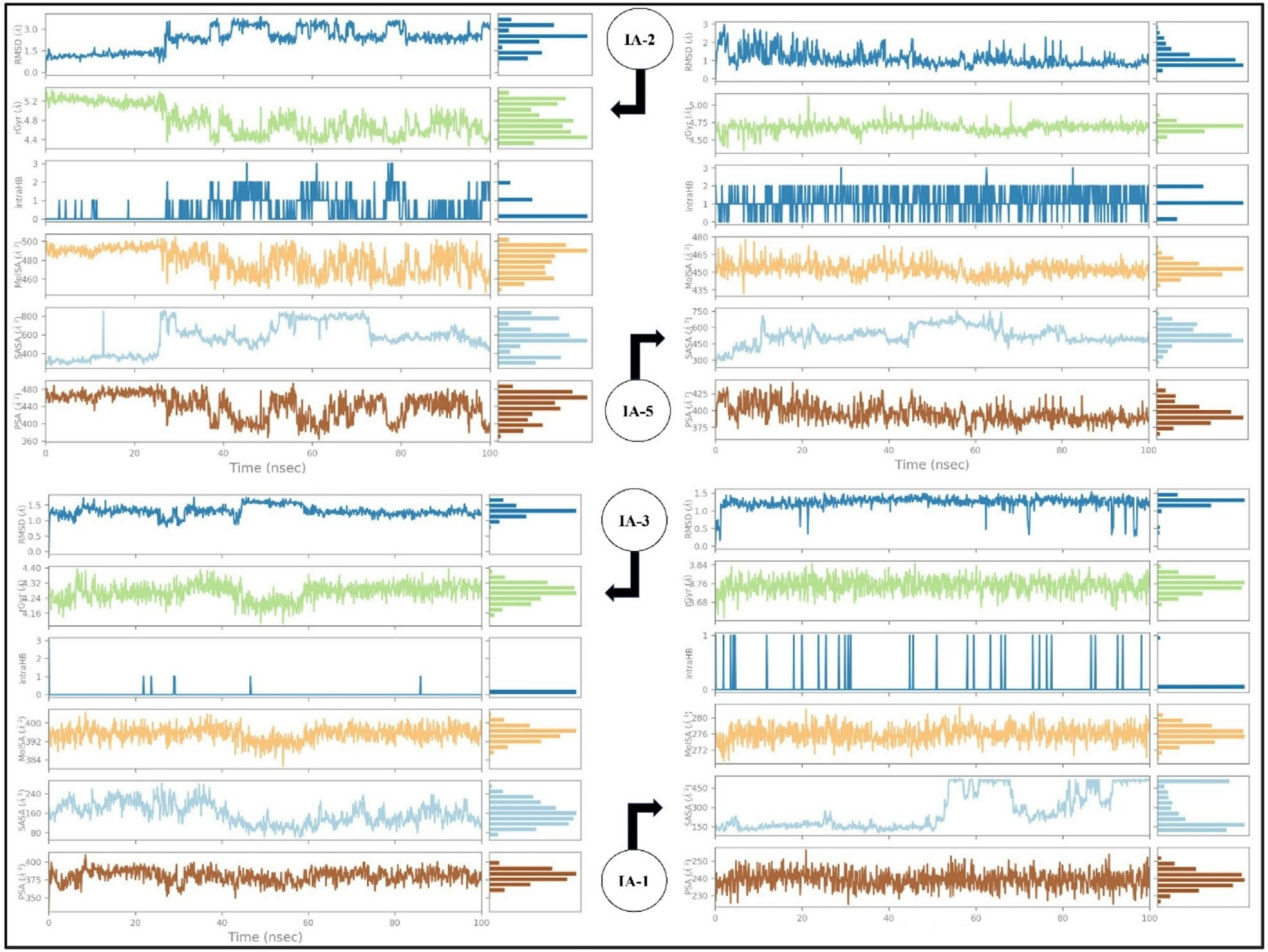
Time-dependent ligand properties of IA-2, IA-5, IA-3, and IA-1 during 100 ns molecular dynamics simulations, including ligand RMSD (Å), radius of gyration (rGyr, Å), intramolecular hydrogen bonds (intraHB), molecular surface area (MolSA, Å²), solvent accessible surface area (SASA, Å²), and polar surface area (PSA, Å²). The x-axis represents simulation time (ns), and each parameter is plotted as a separate trajectory, with legends indicating the respective ligand.

IA-2 exhibited the strongest binding and exceptional dynamic stability over 100 ns, maintaining a low RMSD and a compact radius of gyration with multiple hydrogen bonds observed throughout the trajectory that anchored the ligand in the pocket. Interaction mapping during the trajectory shows sustained contacts with Asp404, Asp518, Asp616, Trp376, and Phe649, while SASA decreased slightly with the binding site remaining largely preserved. IA-5 retained a stable binding pose throughout the 100 ns run, showing modest RMSD fluctuations and a steady Rg consistent with a well-packed complex; hydrophobic and π interactions contributed to long-lived contacts. Key residues Asp282, Trp376, Phe525, Leu405, and Leu677 repeatedly engaged the ligand, H bonding was intermittent but supplemented by persistent hydrophobic burial (reduced local SASA), with minimal structural perturbation observed in the binding region. IA-3 displayed moderate conformational stability with occasional RMSD excursions and transient hydrogen bonds, indicating a dynamic but retained occupancy of the active site over 100 ns. The ligand formed recurrent interactions with Asp282, Trp376, Asp404, and Asp518 and hydrophobic contacts with Phe649 and Phe525; SASA and PSA traces showed short-lived fluctuations consistent with partial flexibility at the binding interface. IA-1 showed the greatest structural variability during the 100 ns simulation, with higher RMSD and increased radius of gyration reflecting looser pocket complementarity and fewer persistent hydrogen bonds. Contacts with Asp282, Trp376, Trp481, Leu405, Asp518, and Asp616 were observed but less stable, accompanied by transient increases in SASA indicating weaker overall stabilization of the complex. Overall, IA 2 exhibited the strongest overall profile with the best docking score and superior dynamic stability (lowest RMSD, compact Rg, consistent hydrogen bonding interactions), IA 5 closely followed with robust hydrophobic and π stacking stabilization, IA 3 showed moderate binding with transient H bonds and occasional RMSD excursions, and IA 1 displayed the weakest pocket complementarity with higher RMSD, increased SASA, and partial structural perturbations over 100 ns (Fig. 16).

The present MD trajectories demonstrate that IA‑2 (rutin) and IA‑5 (kaempferol‑3‑O‑ rhamnoglucoside) maintain the most favorable dynamic profiles rapid equilibration, low backbone and ligand RMSD, compact radius of gyration, sustained hydrogen‑bond networks and persistent hydrophobic/aromatic contacts with conserved active‑site residues such as Asp282, Asp404, Asp518, Asp616, Trp376, Phe525, and Phe649 which mirrors earlier reports where rutin and related flavonoids showed robust docking scores and stable MD behavior against α‑glucosidase/α‑amylase targets (e.g., rutin’s sustained interactions and predicted inhibitory activity reported in computational and biochemical studies). Specifically, prior in‑silico and in‑vitro investigations have documented rutin’s capacity to form multiple H‑bonds and hydrophobic contacts that stabilize its binding and translate into measurable enzyme inhibition, supporting our finding that IA‑2 is a top candidate for follow‑up assays^87,88^. The studies of kaempferol glycosides and other flavonoid scaffolds report comparable docking and MD concordance modest ligand rearrangements followed by strong contacts and reduced local SASA consistent with our IA‑5 trajectories that show intermittent conformational adjustment but overall sustained pocket occupancy^89^. Complementary RMSF and PSA analyses in the literature also note low fluctuations for residues directly engaging flavonoid ligands and preservation of secondary‑structure elements near the binding site, which aligns with our observation of localized loop flexibility but stable active‑site architecture across IA‑2 and IA‑5 complexes^90^. Taken together, these convergent lines of evidence from docking, 100‑ns MD, and previously published computational and experimental studies support prioritizing IA‑2 and IA‑5 for biochemical validation as promising natural α‑glucosidase inhibitors^88,89,91^.

The docking affinities, electronic descriptors and MD stability metrics with published flavonoid α-glucosidase data, underscoring the novel combined DFT and 100-ns MD evidence that prioritizes IA-2 and IA-5 for biochemical validation. However, MM/GBSA rescoring was not performed, and thus the reported docking scores should not be interpreted as quantitative binding free energies.

## Conclusion

In this study, an integrated in silico approach combining molecular docking, DFT, ADMET, and 100 ns molecular dynamics simulations was applied to evaluate the α-glucosidase inhibitory potential of I. aquifolium flavonoids. Docking analysis identified IA-2 (rutin) and IA-5 (kaempferol-3-O-rhamnoglucoside) as strong binders, with binding affinities (–9.59 and − 9.18 kcal/mol) comparable to acarbose (–10.96 kcal/mol), supported by key interactions with catalytic residues such as Asp404, Asp518, Asp616, and Trp376. DFT analysis indicated favorable electronic characteristics across the ligands, with IA-1 showing the highest dipole moment and IA-4 the lowest HOMO–LUMO energy gap, suggesting potential reactivity. ADMET evaluation highlighted IA-1 as the most drug-like compound, with relatively better bioavailability and pharmacokinetic properties, whereas IA-2 and IA-5 exhibited lower predicted bioavailability, indicating possible limitations in oral absorption. MD simulations further supported the docking results by demonstrating stable protein–ligand complexes, where IA-2 and IA-5 showed lower RMSD values, compact structural profiles, and sustained interactions, while IA-3 and IA-1 exhibited comparatively higher flexibility. Overall, this study provides a hypothesis-generating framework that prioritizes IA-2 and IA-5 based on binding stability and IA-1 based on drug-likeness. These findings offer a rational basis for future experimental validation, and the proposed compounds should be further evaluated through in vitro and in vivo studies before considering therapeutic applications.

## Data Availability

All data generated and/or analyzed during this study are included in this article and its Supplementary Information. Specifically, phytochemicals were retrieved from the IMPPAT and Available literature followed by making of structures through ChemDraw Professional (2019) and the three-dimensional structure of the target protein was obtained from the Protein Data Bank (PDB ID: 5NN8). Other software used in the study including, Schrodinger 2020-3 (Maestro 12. 5), BIOVIA Discovery Studio (2025), GuassView, PyMol etc. No new experimental datasets such as proteomics, genomic, transcriptomic, or crystallographic data were generated in this study. Additional computational data and analysis files are available from the corresponding author upon reasonable request.
